# Supplementary material for: The clock in growing hyphae and their synchronization in Neurospora crassa
Source: Commun Biol. 2024 Jun 18;7:735. doi: 10.1038/s42003-024-06429-6 (PMC11189396; doi:10.1038/s42003-024-06429-6)
Supplement: Supplementary file 2 — Supplementary Information [file 42003_2024_6429_MOESM2_ESM.pdf]

Supplementary Materials for

The clock in growing hyphae and their synchronization in *Neurospora crassa*

Authors: Jia Hwei Cheong[1*], Xiao Qiu[2*], Yang Liu[1], Shishir Bhusal[3], Emily Krach[4], Yinping Guo[4], Heinz-Bernd Schüttler[3], Jonathan Arnold[4], and Leidong Mao[5]

[*]Both authors have contributed equally to this work.

Affiliations:

[1]Chemistry Department, University of Georgia, Athens, GA, 30602, USA.

[2]Institute of Bioinformatics, University of Georgia, Athens, GA, 30602, USA.

[3]Department of Physics and Astronomy, University of Georgia, Athens, GA, 30602, USA.

[4]Genetics Department, University of Georgia, Athens, GA, 30602, USA.

[5]School of Electrical and Computer Engineering, College of Engineering, University of Georgia, Athens, GA, 30602, USA.

Corresponding authors: Jonathan Arnold, arnold@uga.edu

This pdf includes:

Supplementary Fig. S1, S2, S3, S4, S5, S6, S7, S8, S9, S10, Supplementary Table S1, S2, S3, S4, S5, S6, S7 and S8.

Other supplementary materials include:

Supplementary Movie S1, Movie S2, Supplementary Data 1, Supplementary Data 2, Supplementary Data 3.

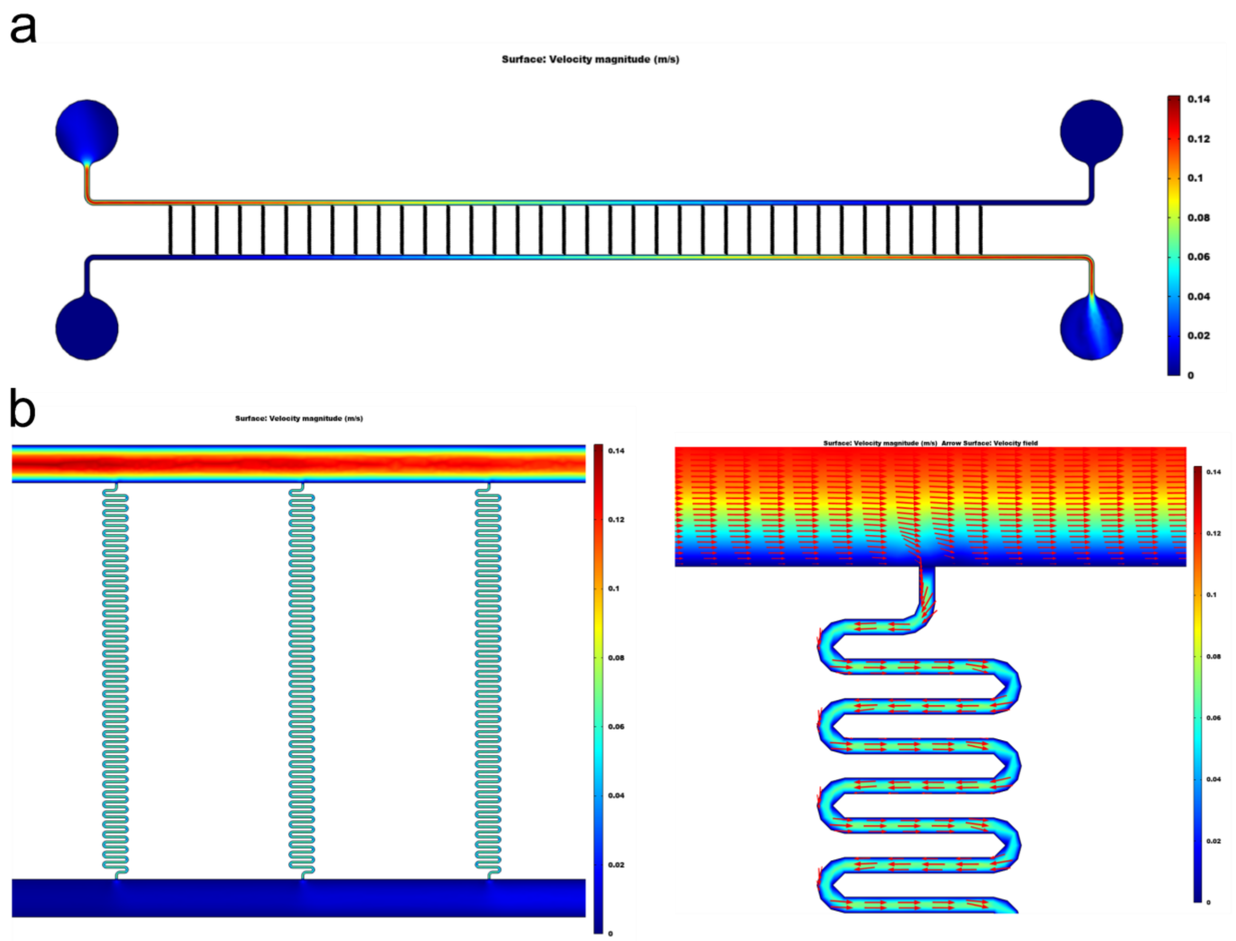

Supplementary Figure S1. Numerical simulation of flow profiles within the channels using COMSOL Multiphysics software. (**a**) Simulated velocity flow profile in the whole serpentine chip shows constant flow of media. The right panel- color bar indicates the velocity. (**b**) (Left) Magnified image of the simulated velocity flow profile in random selected serpentine channels. Flow is present in the serpentine channel. (Right) Magnified image of the velocity profile at the sepentine inlet closest to the media flow.

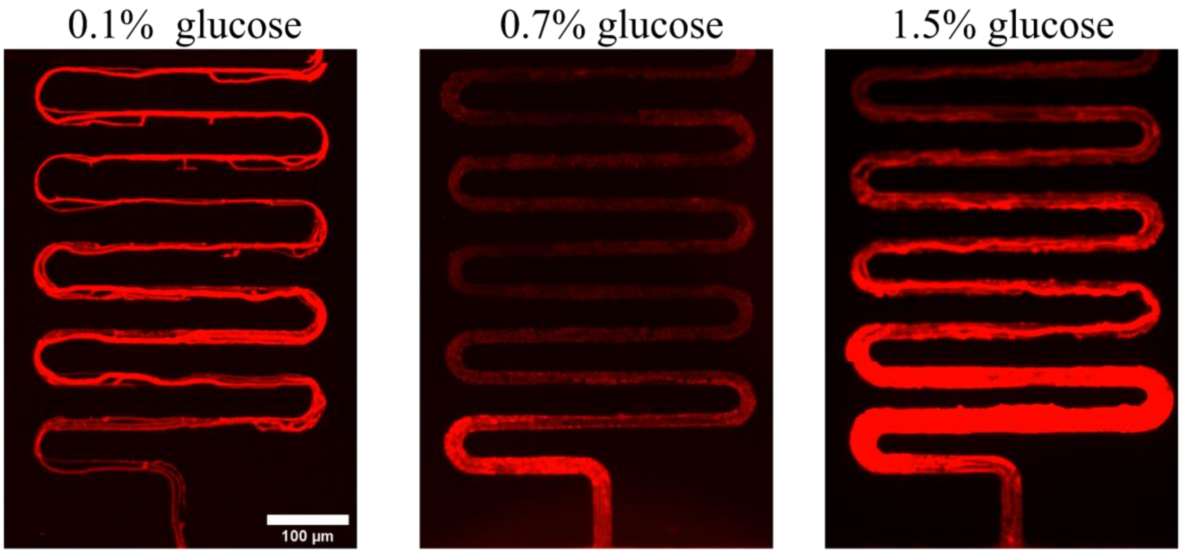

Supplementary Figure S2. Fluorescence images on MFNC9 hyphae near the inlet of serpentine channels with varying glucose (0.1% ,0.7% and 1.5% glucose) growing for 48h. The filaments in serpentine channels increase in thickness with the increase of glucose concentration. Scale bar, 100µm.

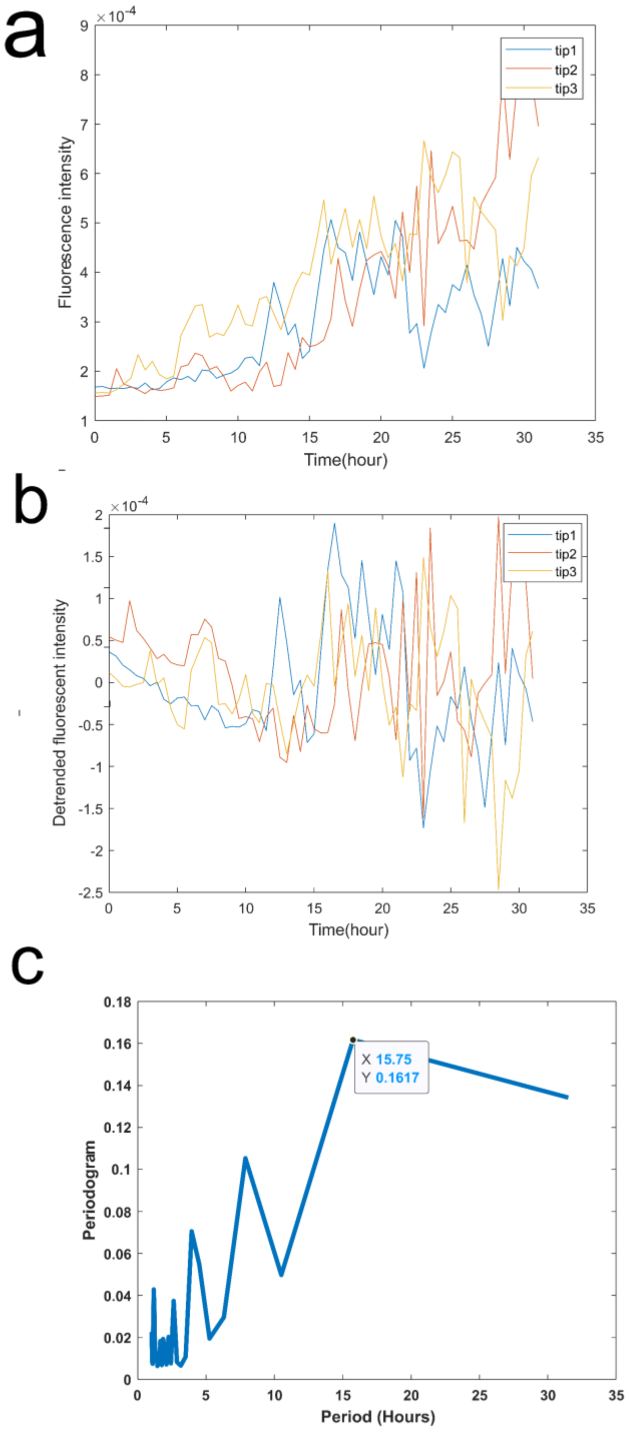

Supplementary Figure S3. MFNC9 fluorescence trajectories of three individual hyphal tips tracked. (**a**) Raw fluorescence trajectories of hyphal tip tracked over a period of 30 hours. (**b**) Detrended fluorescence trajectories of hyphal trip tracked. (**c**) The average periodogram of hyphal tips present a period of 15.75 hours.

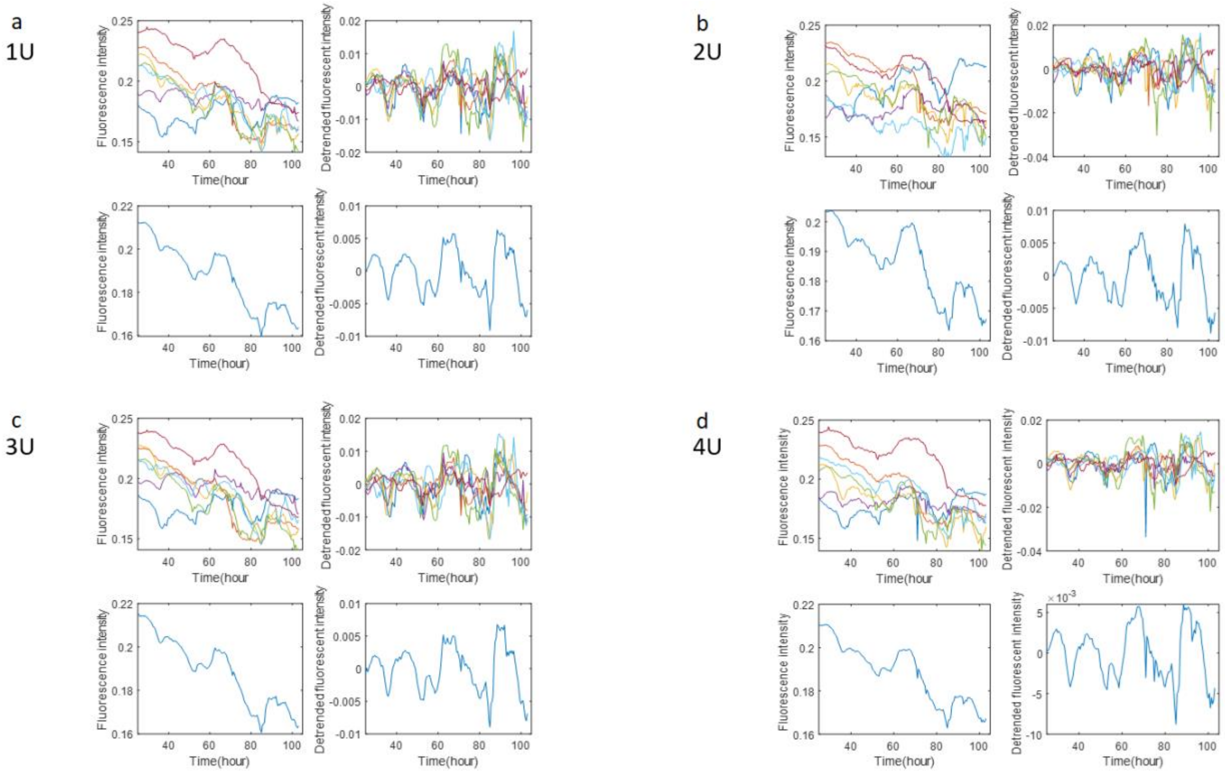

Supplementary Figure S4. MFNC9 fluorescence trajectories of seven individual segments of hyphae in serpentine channels.. (**a-d**) Top left graph show raw fluorescence trajectories of seven individual segments while the top right graph is the detrended trajectories that were carried out with a 24-hour detrending window. The bottom left graph is the average raw fluorescence trajectories while the bottom right graph is the average detrended trajectories. The corresponsing number of U-turns tracked for each graph is labelled in the image.

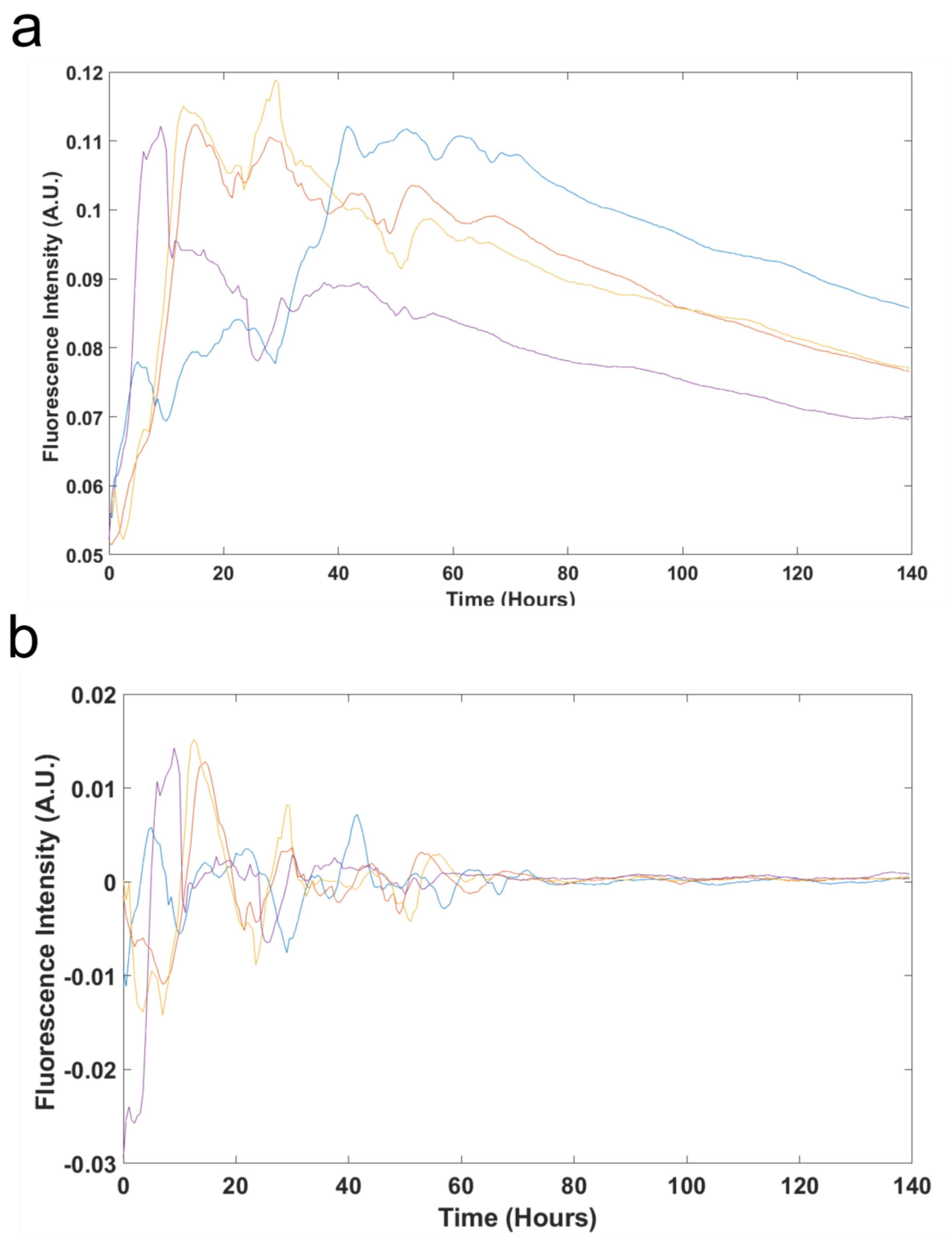

Supplementary Figure S5. MFNC9 fluorescence trajectories of hyphae in four different serpentine channels. (**a**) Raw fluoresence tracjectories of hyphae growing in channels (b) Detrended fluorescene trajectories with a 24-hour detrending window.

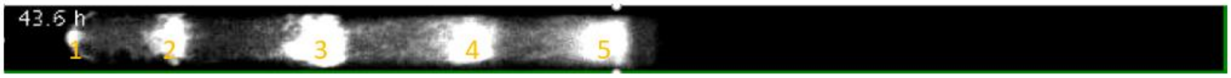

Figure S6. Image of luminescence band in a race tube[1]. Numbers labeled in the image correspond to the bands tracked over time in Figure 6.

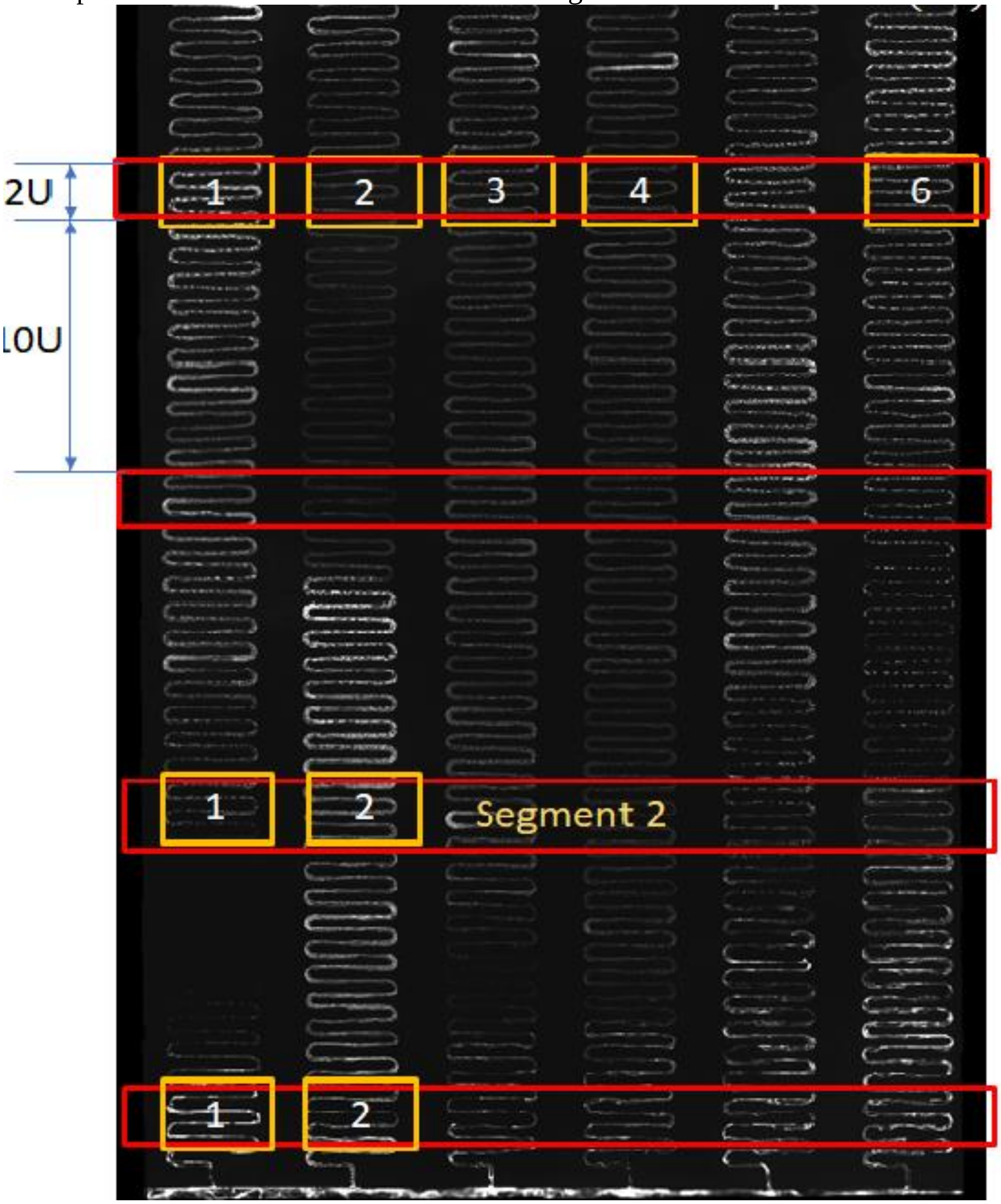

Supplementary Figure S7. Image of the segment (segment 2) consisting of six serpentine channels tracked to calculate Kuramoto K values in Table 2**.**

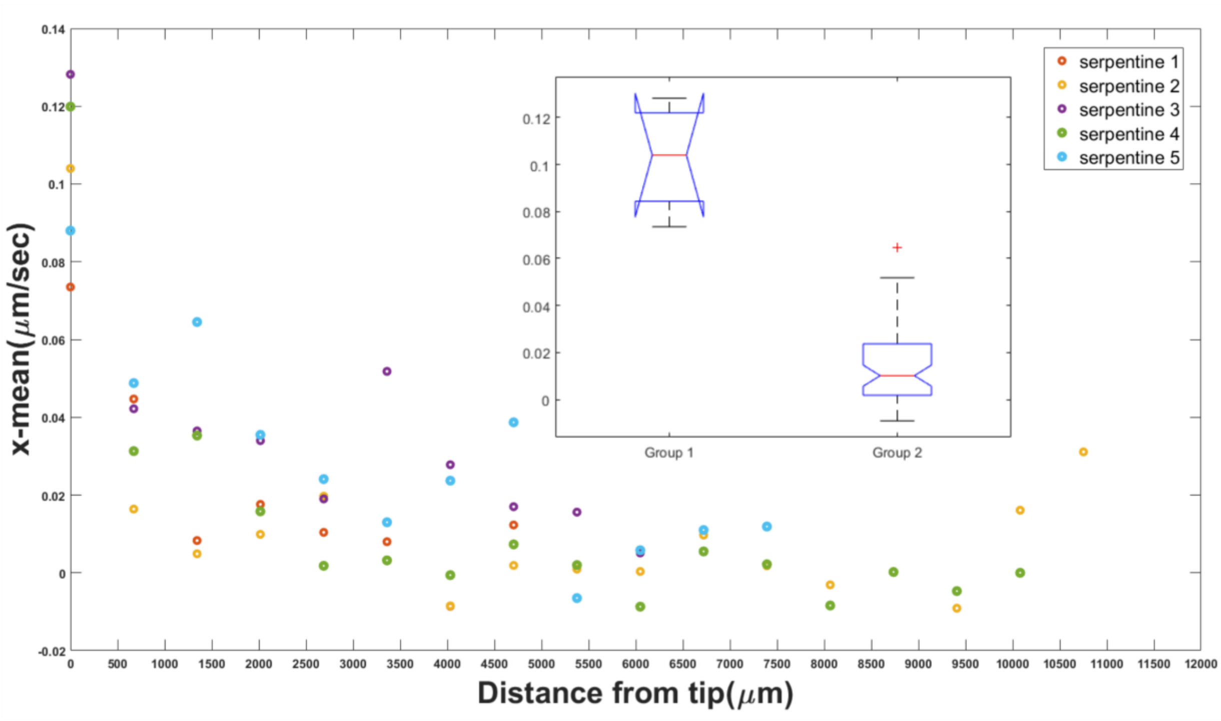

Supplementary Figure S8. The average nuclear drift velocity in 5 varying serpentine channels on one device is plotted vs. the distance from the tip. The x-y coordinates was obtained from the slope obtained using the method described in Fig.10 over 10 sequential images taken at 3 second intervals. Medium was infused into the serpentine device at a constant flow rate of 0.1μm/min. Box plot of the Kruskal-Wallis correlation of the average drift velocity according to the distance from the tip. Group 1 includes the nuclear drift velocity at the tip while for each serpentine channel while Group 2 includes all drift velocity away from the tip.

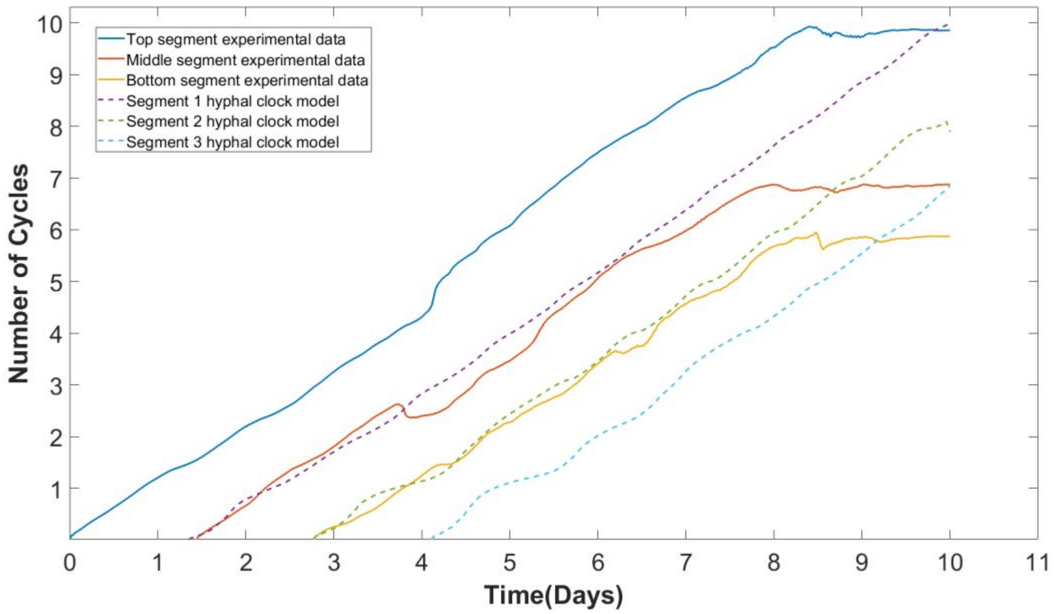

Supplementary Figure S9. Plot of the experimental and model results of the number of cycles vs. real time. We are able to observe a phase shift when it is plotted out on the real time when growth is initiated. The solid lines depict experimental data while the dotted lines correspond to the hyphal clock model results as shown in **Fig. 12**.

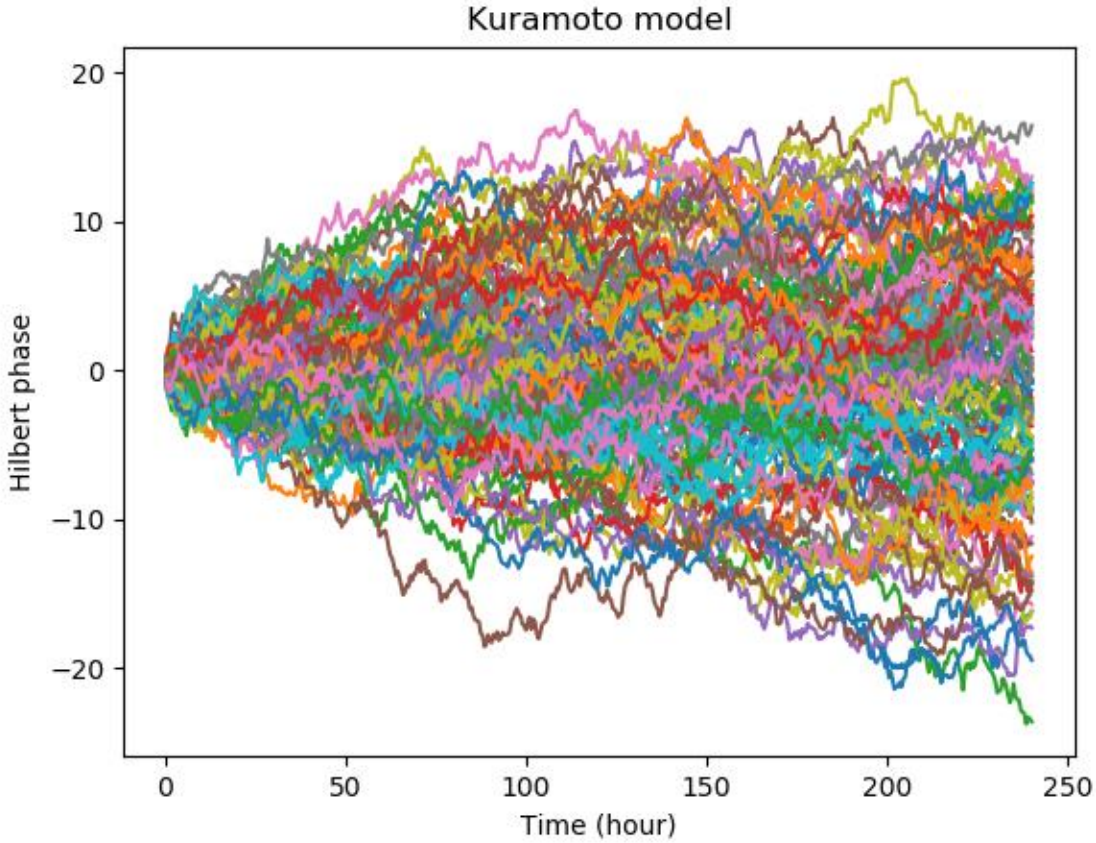

Supplementary Figure S10. Hilbert phase[2] of 127 trajectories of generated white noise for a period of 240 hours. Hilbert phase was plotted to calculate the Kuramoto (K) number.

Replicate	Signal to Noise	Period (h)
1	9.38	23
2	6.52	23
3	2.92	23
4	34.70	23
5	13.55	23
6	3.94	23
7	11.48	17.5
8	21.21	23
Average (+/- standard error)	12.96 (+/-3.73)	22.31(+/-0.69)

Supplementary Table S1. The signal-to-noise-ratio (S/N) for hyphae in 8 different serpentine channels. The S/N was calculated by dividing the mean amplitude of the signal over the noise (see *Materials and Methods*). The FT method was employed.

Temperature (T_1)	24° C	25° C	27° C	29° C
Period (Hours) (+/-SE)	20.00 (+/- 2.0335)	20.00 (+/- 0.4336)	20.00 (+/- 1.4320)	20.000 (+/- 1.8819)
Q_{10} (+/-SE)	1.0167(+/- 0.0369)	1.113(+/- 0.0124)	1.0337(+/- 0.0906)	1.1046(+/- 0.0416)

Supplementary Table S2. Temperature Coefficient Q_{10} over a physiological range of temperatures. T_1 represents the experimental temperature. Standard Errors (SE) for temperature coefficient were computed using the propagation of error method. We used the FT method.

**Two-way ANOVA**	**Sum Sq.**	**d.f.**	**Mean Sq.**	**F**	***p*-value**
**Serpentine channel**	0.00227	4	0.00057	2.59	0.0472
**Distance from tip**	0.03473	1	0.03473	158.04	0
**Serpentine channel * Distance from tip**	0.00211	4	0.00053	2.4	0.0610
**Error**	0.01165	53	0.00022		
**Total**	0.05495	62			

Supplementary Table S3. Two-way analysis of variance (ANOVA) of the dependence of the velocity profile on the serpentine channel and distance from tip.

**Source**	**SS**	**df**	**MS**	**Chi-sq**	***p*-value**
**Distance from tip**	4567.5	1	4567.5	13.59	0.0002
**Error**	16264	61	266.62		
**Total**	20831.5	62			

Supplementary Table S4. Kruskal-Wallis ANOVA of the relationship between velocity and the distance from tip.

Kuramoto K	Serpentine 1	Serpentine 2	Serpentine 3	Serpentine 4	Serpentine 5	Serpentine 6
Serpentine 1	-	-	-	-	-	-
Serpentine 2	0.8272	-	-	-	-	-
Serpentine 3	0.7937	0.7903	-	-	-	-
Serpentine 4	0.7526	0.7866	0.7901	-	-	-
Serpentine 5	0.8239	0.8763	0.8193	0.8196	-	-
Serpentine 6	0.8148	0.8027	0.9045	0.8107	0.8511	-

Supplementary Table S5. Measures of synchronization (K) between 6 different serpentine channels. Measures of synchronization (K) between 6 different serpentine channels were obtained with the ST method (Supplementary Fig. S7) along the chip on MFNC9 hyphae.

	Drift Velocity (μm/min) (+/-SE) Top	Drift Velocity (μm/min) (+/-SE) Mid	Drift Velocity (μm/min) (+/-SE) Bottom
Replicate 1	1.29 (+/-0.03)	1.10 (+/-0.03)	2.62 (+/-0.06)
Replicate 2	1.10 (+/-0.1)	1.62 (+/-0.1)	0.864 (+/-0.1)
Replicate 3	2.72 (+/-0.2)	2.69 (+/-0.2)	3.71(+/-0.2)
Replicate 4	2.51(+/-0.1)	3.49 (+/-0.2)	4.76 (+/-0.2)

Supplementary Table S6. Drift velocity of nuclei in the x-direction calculated for three different sections in four different serpentine channels near the hyphal growth tip.

Supplementary Table S7. Parameters for the hyphal clock model used in the main text.

Value	Parameters
7.08E-11	A
0.1013056	Abar
2.74E-08	Ac
9.52778	Bc
1.6854343	C1
0.0361261	C2
1.19549	D1
1	D2
0.476308	D3
0.5428893	D4
0.370788	D5
0.226692	D6
0.0430166	D7
6.59E-05	D8
60.49845	D_{cr}
0.3940957	D_{cp}
4234.50804	L1
2089.61144	L2
5.283324	L3
12.56038	Lc
24.61307	P
0.284950685	S1
0.003348683	S2
0.001721919	S3
0.194833069	S4
0.597068753	Sc
0.0021	D10/Eta
0.005783136	Eta
21.50455	D9
0.02667788	C4
9.225576	kSi
1.21E-05	D10
0	C3

Supplementary Table S8. Parameters for another model with advection zone width of 0.5 mm. The dynamics are similar to the model in Supplementary Table S4.

Value	Name
7.07861703E-11	A
0.1013056	Abar
2.74347681E-08	Ac
9.52778	Bc
1.6854343	C1
0.01083783	C2
1.19549	D1
1	D2
0.476308	D3
0.5428893	D4
0.370788	D5
0.226692	D6
0.0430166	D7
6.586861E-05	D8
60.49845	Dcr
0.3940957	Dcp
8.46901609	L1
2089.61144	L2
5.283324	L3
12.56038	Lc
7.8761824	P
0.284950685	S1
0.0033486825	S2
0.00172191948	S3
0.194833069	S4
0.597068753	Sc
0.0021	D10/Eta
0.005783136	Eta

21.50455	D9
0.0266778796	C4
9.225576	kSi
1.21445856E-05	D10
0.00541891498	C3

Parameter set for the model with 0.5 mm advection width.

References

1 Gooch, V. D. *et al.* Fully codon-optimized luciferase uncovers novel temperature characteristics of the Neurospora clock. *Eukaryot Cell* **7**, 28-37 (2008).

2 Caranica, C. *et al.* What is Phase in Cellular Clocks? *Yale J Biol Med* **92**, 169-178 (2019).
