# Supplementary material for: The clock in growing hyphae and their synchronization in Neurospora crassa
Source: Commun Biol. 2024 Jun 18;7:735. doi: 10.1038/s42003-024-06429-6 (PMC11189396; doi:10.1038/s42003-024-06429-6)
Supplement: Supplementary file 3 — Description of Additional Supplementary Files [file 42003_2024_6429_MOESM3_ESM.docx]

**Description of Additional Supplementary Files**

File name: Supplementary Movie S1.
Description: Video of the strain (N2281-3) with a fluorescent recorder on the H1 Histone imaged every 5 seconds over 10 minutes to observe the nuclei movement at the hyphal tip.

File name: Supplementary Movie S2.
Description: Video of the strain (N2281-3) with a fluorescent recorder on the H1 Histone imaged every 3 seconds over 3 minutes to observe the nuclei movement at the entrance of a serpentine channel after the hyphal tip have elongated for a period of 3 days.

File name: Supplementary Data 1

Description: Zip file containing matlab code for model.

File name: Supplementary Data 2

Description: Manual for the Hyphal Clock Model.

File name: Supplementary Data 3

Description: A full description of the Hyphal Clock Model.
