# Supplementary material for: The clock in growing hyphae and their synchronization in Neurospora crassa
Source: Commun Biol. 2024 Jun 18;7:735. doi: 10.1038/s42003-024-06429-6 (PMC11189396; doi:10.1038/s42003-024-06429-6)
Supplement: Supplementary file 7 — Supplementary Data 2 [file 42003_2024_6429_MOESM7_ESM.pdf]

Clock Tube Model: MatLab Code Documentation
V.200525-1704hbs

A. Model Equations and Discretization

The Clock Tube Model is a partial differential equation (PDE) system, describing the spatio-temporal evolution of a multi-nuclear clock system that is undergoing 1D spatial growth of its filamental cellular domain. The PDE system model may be applicable to the 1D growth of a *N. crassa* cell population into a race tube, or to the growth of *N. crassa* fungal hyphae into microdevice channels. The growth of the intra-cellular hyphal filament volume is driven by an uptake of liquid matter (mostly water?) from the extra-cellular space, near the hyphal growth front, accompanied by the advection (drift) of intra-cellular matter towards the hyphal growth front.

Dynamical Variables. Molecular species concentrations and other space-time-dependent dynamical variables of the PDE model are denoted by $Y_k(x,t)$ where k is an integer index, t is time and x is the spatial coordinate, defined on a domain of length L, with

$$0 \;\leq\; x \;\leq\; L \; . \tag{1}$$

The PDE system for these dynamcical variables, described below, is solved on a finite time domain, of duration T, such that

$$0 \;\leq\; t \;\leq\; T \; . \tag{2}$$

At time $t = 0$, user-supplied initial conditions (ICs), $Y_k^{(\text{ini})}(x)$, are imposed, for all k and x, by setting

$$Y_k(x,0) \;=\; Y_k^{(\text{ini})}(x) \; . \tag{3}$$

The index $k = 1, 2, ...K$ is used internally in the code to enumerate the K dynamical variables, $Y_k(x,t)$, evolving in time and space according to the PDE system. These variables include $K-7$ "standard" intracellular molecular species in the clock kinetic rate equation system, with the intra-cellular signal, Si, enumerated as $[Si](x,t) \equiv Y_{K-7}(x,t)$. The remaining seven dynamical variables, *i.e.*, $Y_k(x,t)$ with $k = K-6, K-5, ..., K$, are explained in the following.

In addition to the $K-7$ molecular species, we have to introduce six new intracellular dynamical variables into the model in order to describe the effects of mitotic nuclear division, and its dependence on an ambient nutrient supply. Specifically, we need to keep track of how nuclear division processes affect (sub-)populations of nuclei with different gene activation states. To that end, let us denote the four possible activation states of the (frq, ccg) gene pair in each nucleus by

$$00 \;=\; frq = \text{OFF}, \quad ccg = \text{OFF} \tag{4}$$
$$01 \;=\; frq = \text{OFF}, \quad ccg = \text{ON} \tag{5}$$
$$10 \;=\; frq = \text{ON}, \quad ccg = \text{OFF} \tag{6}$$
$$11 \;=\; frq = \text{ON}, \quad ccg = \text{ON} \tag{7}$$

Copyright © 2020 University of Georgia. Unauthorized duplication or distribution prohibited.

The state "ON" indicates a frq- or ccg-gene whose WCC-activator binding site is occupied by the full complement of n WCC-molecules; "OFF" indicates the binding site to be unoccupied. Here n denotes the cooperativity exponent of the gene activation. Notice that we are assuming here that each nucleus contains exactly *one copy* of the frq-gene and *one copy* of the ccg.

We can then partition the population of all nuclei into four "gene-state resolved" sub-populations, based on the foregoing four activation states, with partial densities of these four sub-populations denoted, resp., by

$$\rho_{00}(x,t)\ ,\quad \rho_{01}(x,t)\ ,\quad \rho_{10}(x,t)\ ,\quad \rho_{11}(x,t)\ . \tag{8}$$

The total nuclear density, denoted by $\rho(x,t)$, is then given by

$$\rho(x,t)\ =\ \rho_{00}(x,t)\ +\ \rho_{01}(x,t)\ +\ \rho_{10}(x,t)\ +\ \rho_{11}(x,t)\ . \tag{9}$$

The PDEs describing the time-evolution of these nuclear density variables and their coupling to the intra-cellular clock dynamics is described below.

In addition, we want to keep track of, potentially, two extra-cellular species: the concentrations of the extra-cellular signal, $[Se](x,t)$, and an extra-cellular nutrient concentration, denoted by $\phi(x,t)$. The latter can be used to model possible effects of local nutrient availability and nutient exhaustion on the mitotic growth of the local nuclear population.

In our enumeration of dynamical variables, $Y_k(x,t)$, the foregoing seven species are then included as follows:

$$\rho_{00}(x,t)\ \equiv\ Y_{K-6}(x,t) \tag{10}$$
$$\rho_{01}(x,t)\ \equiv\ Y_{K-5}(x,t) \tag{11}$$
$$\rho_{10}(x,t)\ \equiv\ Y_{K-4}(x,t) \tag{12}$$
$$\rho_{11}(x,t)\ \equiv\ Y_{K-3}(x,t) \tag{13}$$
$$\rho(x,t)\ \equiv\ Y_{K-2}(x,t) \tag{14}$$
$$[Se](x,t)\ \equiv\ Y_{K-1}(x,t) \tag{15}$$
$$\phi(x,t)\ \equiv\ Y_{K}(x,t) \tag{16}$$

For the specific model presently implented in the code, the complete enumeration by species name is tabulated in the Appendix: there are $K-7\ =\ 17$ intra-cell molecular species and hence

$$K\ =\ 24\ . \tag{17}$$

Based on the foregoing assumption that each nucleus contains exactly *one copy* of the frq-gene and *one copy* of the ccg, the total and partial nuclear densities are related to concentrations of frq^0, frq^1, ccg^0 and ccg^1 by the following "sum rules":

$$[frq^0](x,t)\ =\ \rho_{00}(x,t)\ +\ \rho_{01}(x,t) \tag{18}$$
$$[frq^1](x,t)\ =\ \rho_{10}(x,t)\ +\ \rho_{11}(x,t) \tag{19}$$

Copyright © 2020 University of Georgia. Unauthorized duplication or distribution prohibited.

$$
\begin{aligned}
[ccg^0](x,t) &= \rho_{00}(x,t) + \rho_{10}(x,t) & (20)\\
[ccg^1](x,t) &= \rho_{01}(x,t) + \rho_{11}(x,t) & (21)\\
\rho(x,t) &= [frq^0](x,t) + [frq^1](x,t) & (22)\\
\rho(x,t) &= [ccg^0](x,t) + [ccg^1](x,t)\ . & (23)
\end{aligned}
$$

It principle, it would be sufficient to include only the partial nuclear densities, $\rho_{00}(x,t)$, $\rho_{01}(x,t)$, $\rho_{10}(x,t)$ and $\rho_{11}(x,t)$, as dynamical variables in the PDE system to be solved, but not the total nuclear density or the frq- and ccg-gene concentrations. One could then use the foregoing Eqs. 18-22 to calculate the gene concentrations, $[frq^0](x,t)$, $[frq^1](x,t)$, $[ccg^0](x,t)$ and $[ccg^1](x,t)$ and the total nuclear density, $\rho(x,t)$, from the numerical PDE solutions for the partial nuclear densities. However, in our PDE solver code, we have *not* followed this approach. Instead, as a check of the numerical accuracy of our PDE solver code, we actually *do include* total nuclear density or frq- and ccg-gene concentrations as dynamical variables in our numerical PDE solution, in addition to the partial nuclear densities. We then use numerical solution compliance with Eqs. 18-23 as a test of our numerical solution accuracy.

Partial Differential Equation System. The coupled PDE system for the $K-7$ intra-cellular molecular species has the general form

$$
\partial_t Y_k(x,t) = R_k(\hat{Y}(x,t),t) - \partial_x \Big(v(x,t)\, Y_k(x,t)\Big) \qquad \text{for} \quad k = 1,2,...,K-7 \tag{24}
$$

where $\hat{Y}(x,t)$ is short-hand for

$$
\hat{Y}(x,t) := [Y_1(x,t),\ ...,\ Y_K(x,t)]\ . \tag{25}
$$

Here, $R_k(\hat{Y},t)$ denotes the intra-celluar clock rate function for intra-cell species Y_k, as described in our earlier publications, and in more detail in the Appendix. Note that R_k acquires an explicit time dependence, *i.e.*, for certain species, k, R_k is a fct of both $\hat{Y}$ *and* of time t, if the clock system is subjected to time-dependent external perturbations, such as a time-varying exposure to light. Mitotic nuclear division processes also contribute to the clock rate functions, R_k, of several intra-cell molecular species, specifically, to the active and inactive gene species, frq^0, frq^1, ccg^0 and ccg^1, and, for certain model versions, to the gene activator WCC. These rate function modifications, due to light exposure and nuclear division, are also described in detail in the Appendix.

The term involving the intra-cellular advection (drift) velocity profile, denoted by $v(x,t)$, describes the advection (drift) of intra-cellular matter towards the filamental growth front, due to the uptake of matter from the extra-cellular space in the vicinity of the growth front. The velocity profile, $v(x,t)$, must be provided by the user, as an input into the model.

The rate function R_{K-7}, for the intra-cellular signal, $[Si] \equiv Y_{K-7}$, includes the usual Si-degradation term and the usual cross-membrane diffusion terms, describing the transport of Si into the extra-cellular volume, and the transport of Se from the extra-cellular into the intra-cellular volume, as given in Strogatz *et al.* and used in our earlier work. In addition to advection, we also allow for the possibility of Si being transported by 1D diffusion, within

Copyright © 2020 University of Georgia. Unauthorized duplication or distribution prohibited.

the intra-cellular space, along the filament direction, with a diffusion coefficient Δ_{Si}:

$$\partial_t\,[Si](x,t) \;=\; k_{\mathrm{Si}}\,[CCG](x,t) \;-\; D_9\,[Si](x,t) \;+\; k_{\mathrm{ICM}}\Big([Se](x,t) \;-\; [Si](x,t)\Big) \\ \;+\; \Delta_{Si}\,\partial_x^2\,[Si](x,t) \;-\; \partial_x\Big(v(x,t)\;[Si](x,t)\Big)\;. \tag{26}$$

where k_{ICM} is the intra-cellular cross-membrane (ICM) diffusion rate coefficent, denoted by "η" in Strogatz *et al.*:

$$k_{\mathrm{ICM}} \;\equiv\; \eta \tag{27}$$

We do *not* employ the quasi-stationary approximation of Strogatz *et al.* for the extra-cellular signal, Se. Rather, $[Se](x,t) \equiv Y_{K-1}(x,t)$ is treated as a dynamical variable which evolves subject to the PDE system, again by a modification of the kinetic rate equations of Strogatz *et al.*, also used in our earlier work. We also allow for the possibility of 1D diffusion of Se in the extra-cellular space, along the filament direction, with a diffusion coefficient Δ_{Se}, resulting in:

$$\partial_t\,[Se](x,t) \;=\; -\,D_{10}\,[Se](x,t) \;+\; k_{\mathrm{XCM}}\Big([Si](x,t) \;-\; [Se](x,t)\Big) \;+\; \Delta_{Se}\,\partial_x^2\,[Se](x,t)\;. \tag{28}$$

where k_{XCM} is the extra-cellular cross-membrane (XCM) diffusion rate coefficent, given by

$$k_{\mathrm{XCM}} \;=\; \frac{\mathcal{V}_{\mathrm{int}}}{\mathcal{V}_{\mathrm{ext}}}\;k_{\mathrm{ICM}}\;. \tag{29}$$

Here, $\mathcal{V}_{\mathrm{int}}$ and $\mathcal{V}_{\mathrm{ext}}$ denote the total intra-cellular volume and the total extra-cellular volume, resp. The PDEs for the total and partial nuclear densities, ρ, ρ_{00}, ρ_{01}, ρ_{10} and ρ_{11}, and for the extra-cellular nutrient, ϕ, are given by

$$\begin{aligned}
\partial_t\,\rho_{00}(x,t) \;=\;& -\,(A+A_c)\;\Omega(x,t)\;\rho_{00}(x,t) \;+\; \bar{A}\;\rho_{10}(x,t) \;+\; B_c\;\rho_{01}(x,t) \\
&+\; k_{\mathrm{ND}}\,\Big(\rho_{00}(x,t) \;+\; 2\rho_{01}(x,t) \;+\; 2\rho_{10}(x,t) \;+\; 2\rho_{11}(x,t)\Big) \\
&\qquad\times\;\phi(x,t)\,\Big(1-(\rho(x,t)/\rho_{\,\mathrm{Lim}})^{\epsilon_\rho}\Big) \\
&-\;\partial_x\Big(v(x,t)\,\rho_{00}(x,t)\Big) && (30)\\
\partial_t\,\rho_{01}(x,t) \;=\;& -\,(A\;\Omega(x,t)+B_c)\;\rho_{01}(x,t) \;+\; \bar{A}\;\rho_{11}(x,t) \;+\; A_c\;\Omega(x,t)\;\rho_{00}(x,t) \\
&-\; k_{\mathrm{ND}}\,\phi(x,t)\,\rho_{01}(x,t)\;\Big(1-(\rho(x,t)/\rho_{\,\mathrm{Lim}})^{\epsilon_\rho}\Big) \\
&-\;\partial_x\Big(v(x,t)\,\rho_{01}(x,t)\Big) && (31)\\
\partial_t\,\rho_{10}(x,t) \;=\;& -\,(\bar{A}+A_c\;\Omega(x,t))\;\rho_{10}(x,t) \;+\; A\;\Omega(x,t)\;\rho_{00}(x,t) \;+\; B_c\;\rho_{11}(x,t) \\
&-\; k_{\mathrm{ND}}\,\phi(x,t)\,\rho_{10}(x,t)\;\Big(1-(\rho(x,t)/\rho_{\,\mathrm{Lim}})^{\epsilon_\rho}\Big) \\
&-\;\partial_x\Big(v(x,t)\,\rho_{10}(x,t)\Big) && (32)\\
\partial_t\,\rho_{11}(x,t) \;=\;& -\,(\bar{A}+B_c)\;\rho_{11}(x,t) \;+\; (A\;\rho_{01}(x,t) \;+\; A_c\;\Omega(x,t)\;\rho_{10}(x,t))\;\Omega(x,t) \\
&-\; k_{\mathrm{ND}}\,\phi(x,t)\,\rho_{11}(x,t)\;\Big(1-(\rho(x,t)/\rho_{\,\mathrm{Lim}})^{\epsilon_\rho}\Big) \\
&-\;\partial_x\Big(v(x,t)\,\rho_{11}(x,t)\Big) && (33)\\
\partial_t\,\rho(x,t) \;=\;& \quad k_{\mathrm{ND}}\,\phi(x,t)\,\rho(x,t)\;\Big(1-(\rho(x,t)/\rho_{\,\mathrm{Lim}})^{\epsilon_\rho}\Big) \;-\; \partial_x\Big(v(x,t)\,\rho(x,t)\Big) && (34)\\
\partial_t\,\phi(x,t) \;=\;& -\,k_{\mathrm{FC}}\,\phi(x,t)\,\rho(x,t)\;\Big(1-(\rho(x,t)/\rho_{\,\mathrm{Lim}})^{\epsilon_\rho}\Big)\;. && (35)
\end{aligned}$$

Copyright © 2020 University of Georgia. Unauthorized duplication or distribution prohibited.

Here, "ND" stands for "nuclear division" and "FC" stands for "food consumption". The factor $\Omega(x,t)$ is shorthand for

$$\Omega(x,t) \;=\; ([WCC](x,t))^n \tag{36}$$

and n is the cooparativity exponent for WCC binding to both the frq and ccg activator binding sites. The parameter ρ_{Lim} allows to impose an upper limit on the mitotic growth of the nuclear density, indepently of nutrient availability. Note that the extra-cellular species, Se and ϕ, are *not* subject to advection: their rate fcts do not contain the $v(x,t)$-term.

From Eqs. 18-21 and Eqs. 30-33 follow the rate equations for the gene concentrations $[frq^0]$, $[frq^1]$, $[ccg^0]$ and $[ccg^1]$:

$$\begin{aligned}
\partial_t\,[frq^0](x,t) \;=\;& -\,A\,\Omega(x,t)\,[frq^0](x,t) \;+\; \bar{A}\,[frq^1](x,t) \\
&+\,k_{\text{ND}}\left([frq^0](x,t) \;+\; 2[frq^1](x,t)\right) \\
&\qquad\times\;\phi(x,t)\left(1-(\rho(x,t)/\rho_{\text{Lim}})^{\epsilon_\rho}\right) \\
&-\,\partial_x\left(v(x,t)\,[frq^0](x,t)\right) \qquad (37)\\
\partial_t\,[frq^1](x,t) \;=\;& +\,A\,\Omega(x,t)\,[frq^0](x,t) \;-\; \bar{A}\,[frq^1](x,t) \\
&-\,k_{\text{ND}}\,\phi(x,t)\,[frq^1](x,t)\,\left(1-(\rho(x,t)/\rho_{\text{Lim}})^{\epsilon_\rho}\right) \\
&-\,\partial_x\left(v(x,t)\,[frq^1](x,t)\right) \qquad (38)\\
\partial_t\,[ccg^0](x,t) \;=\;& -\,A_c\,\Omega(x,t)\,[ccg^0](x,t) \;+\; B_c\,[ccg^1](x,t) \\
&+\,k_{\text{ND}}\left([ccg^0](x,t) \;+\; 2[ccg^1](x,t)\right) \\
&\qquad\times\;\phi(x,t)\left(1-(\rho(x,t)/\rho_{\text{Lim}})^{\epsilon_\rho}\right) \\
&-\,\partial_x\left(v(x,t)\,[ccg^0](x,t)\right) \qquad (39)\\
\partial_t\,[ccg^1](x,t) \;=\;& -\,B_c\,[ccg^1](x,t) \;+\; A_c\,\Omega(x,t)\,[ccg^0](x,t) \\
&-\,k_{\text{ND}}\,\phi(x,t)\,[ccg^1](x,t)\,\left(1-(\rho(x,t)/\rho_{\text{Lim}})^{\epsilon_\rho}\right) \\
&-\,\partial_x\left(v(x,t)\,[ccg^1](x,t)\right) \qquad (40)
\end{aligned}$$

where $\Omega(x,t)$ is given by Eq. 36. Notice that the first two terms, involving A, $\bar{A}$, A_c or B_c in each of the foregoing four equations, are just the usual rates for the gene activation and de-activation processes.

Advection Velocity Profile. The velocity profile, $v(x,t)$, is modeled by

$$v(x,t) \;=\; V_{\text{p}}\,(x - x_{\text{p}}(t)) \tag{41}$$

where $x_{\text{p}}(t)$ is the moving growth front position, assumed to advance with a constant growth speed, u_{p}:

$$x_{\text{p}}(t) \;=\; x_{\text{p0}} \;+\; u_{\text{p}}\,t\;. \tag{42}$$

and ξ is the position coordinate measured relative to the moving growth front:

$$\xi \;=\; x - x_{\text{p}}(t)\;. \tag{43}$$

Copyright © 2020 University of Georgia. Unauthorized duplication or distribution prohibited.

The advection profile shape function, V_p, has the functional form

$$V_\mathrm{p}(\xi) \;=\; u_\mathrm{a}\; f_{v,\mathrm{lo}}(\xi)\; f_{v,\mathrm{hi}}(\xi) \tag{44}$$

where u_a is the (near-)maximal advection velocity in the advection profile. The dimensionless factors $f_{v,\mathrm{lo}}(\xi)$ and $f_{v,\mathrm{hi}}(\xi)$ denote, respectivly, an upward and a downward soft-edge step function for which the code implements the following two options:

Advection Velocity Profile Option 1: Piecewise Cubic Step Functions

$$f_{v,\mathrm{lo}}(\xi) \;=\; \begin{cases} 0 & \text{for } \xi \;\le\; \xi_\mathrm{lo} - W_{v,\mathrm{lo}}/2 \\ q_\mathrm{c}\!\left(\frac{\xi-\xi_\mathrm{lo}}{W_{v,\mathrm{lo}}}\right) & \text{for } \xi_\mathrm{lo} - W_{v,\mathrm{lo}}/2 \;\le\; \xi \;\le\; \xi_\mathrm{lo} + W_{v,\mathrm{lo}}/2 \\ 1 & \text{for } \xi_\mathrm{lo} + W_{v,\mathrm{lo}}/2 \;\le\; \xi \end{cases} \tag{45}$$

$$f_{v,\mathrm{hi}}(\xi) \;=\; \begin{cases} 0 & \text{for } \xi \le \xi_\mathrm{hi} - W_{v,\mathrm{hi}}/2 \\ q_\mathrm{c}\!\left(\frac{\xi_\mathrm{hi}-\xi}{W_{v,\mathrm{hi}}}\right) & \text{for } \xi_\mathrm{hi} - W_{v,\mathrm{hi}}/2 \le \xi \le \xi_\mathrm{hi} + W_{v,\mathrm{hi}}/2 \\ 1 & \text{for } \xi \ge \xi_\mathrm{hi} + W_{v,\mathrm{hi}}/2 \end{cases} . \tag{46}$$

Here q_c is a cubic polynomial, given by:

$$q_\mathrm{c}(\beta) \;=\; -2\;\beta^3 \;+\; \frac{3}{2}\,\beta \;+\; \frac{1}{2} \tag{47}$$

where

$$\beta \equiv \frac{\xi-\xi_\mathrm{lo}}{W_{v,\mathrm{lo}}}\;, \qquad \text{for} \quad f_{v,\mathrm{lo}}(\xi)\;, \qquad\qquad \text{and} \qquad\qquad \beta \equiv \frac{\xi_\mathrm{hi}-\xi}{W_{v,\mathrm{hi}}}\;, \qquad \text{for} \quad f_{v,\mathrm{hi}}(\xi)\;. \tag{48}$$

Note that $q_\mathrm{c}(\beta)$ has the properties

$$q_\mathrm{c}\Big(-\frac{1}{2}\Big) = 0\;, \quad q_\mathrm{c}'\Big(-\frac{1}{2}\Big) = 0\;, \quad q_\mathrm{c}(0) = \frac{1}{2}\;, \quad q_\mathrm{c}\Big(+\frac{1}{2}\Big) = 1\;, \quad q_\mathrm{c}'\Big(+\frac{1}{2}\Big) = 0\;. \tag{49}$$

Consequently, $V_\mathrm{p}(\xi)$ is continuous and differentiable everywhere and it takes on the values

$$V_\mathrm{p}(\xi) \;=\; \begin{cases} 0 & \text{for } \xi \;\le\; \xi_\mathrm{lo} - W_{v,\mathrm{lo}}/2 \\ u_\mathrm{a}/2 & \text{for } \xi \;=\; \xi_\mathrm{lo} \\ u_\mathrm{a} & \text{for } \xi_\mathrm{lo} + W_{v,\mathrm{lo}}/2 \;\le\; \xi \;\le\; \xi_\mathrm{lo} - W_{v,\mathrm{hi}}/2 \\ u_\mathrm{a}/2 & \text{for } \xi \;=\; \xi_\mathrm{hi} \\ 0 & \text{for } \xi_\mathrm{hi} + W_{v,\mathrm{hi}}/2 \;\le\; \xi \end{cases} \tag{50}$$

provided that the two edge locations, ξ_lo and ξ_hi are chosen sufficiently far apart, such that

$$\xi_\mathrm{hi} - \xi_\mathrm{lo} \;\ge\; \frac{W_{v,\mathrm{lo}} + W_{v,\mathrm{hi}}}{2} \tag{51}$$

The functions $f_{v,\mathrm{lo}}(\xi)$, $f_{v,\mathrm{hi}}(\xi)$ and $V_\mathrm{p}(\xi)$, plotted *vs.* ξ, are shown below in Figs.1-4.

Copyright © 2020 University of Georgia. Unauthorized duplication or distribution prohibited.

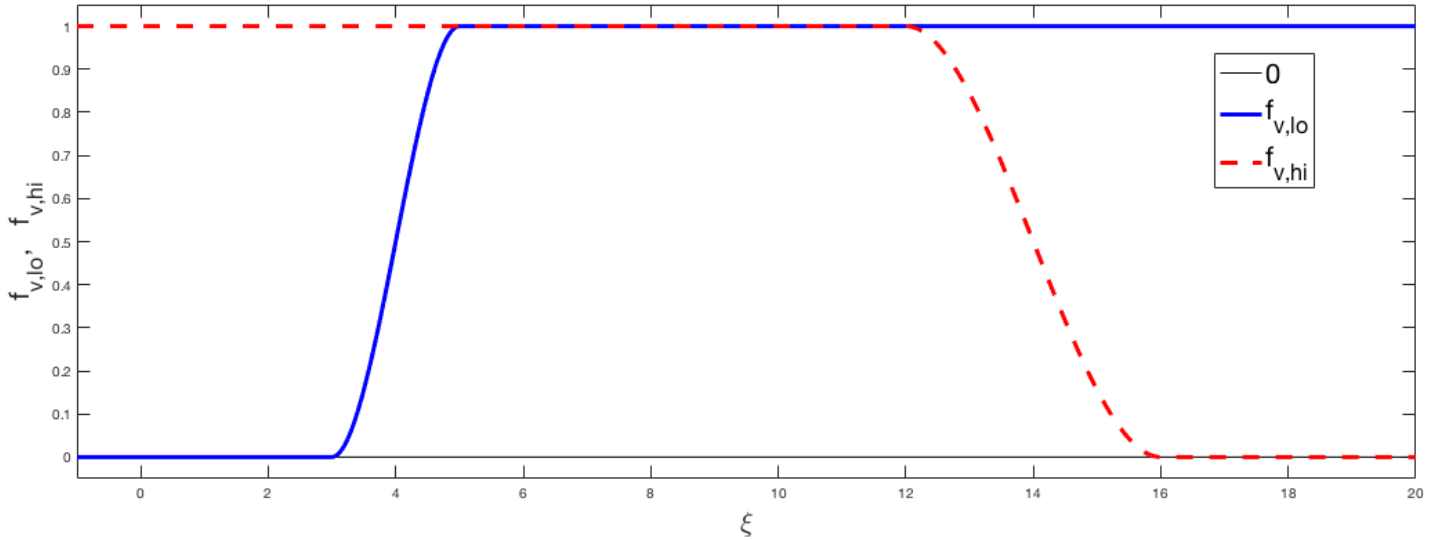

Figure 1. Soft-edge step functions, $f_{v,\mathrm{lo}}(\xi)$ and $f_{v,\mathrm{hi}}(\xi)$, from Option 1 Eqs. 45-48, plotted *vs.* ξ, for edge and width parameters $\xi_{\mathrm{lo}} = 4$, $W_{v,\mathrm{lo}} = 2$, $\xi_{\mathrm{hi}} = 14$, $W_{v,\mathrm{hi}} = 4$.

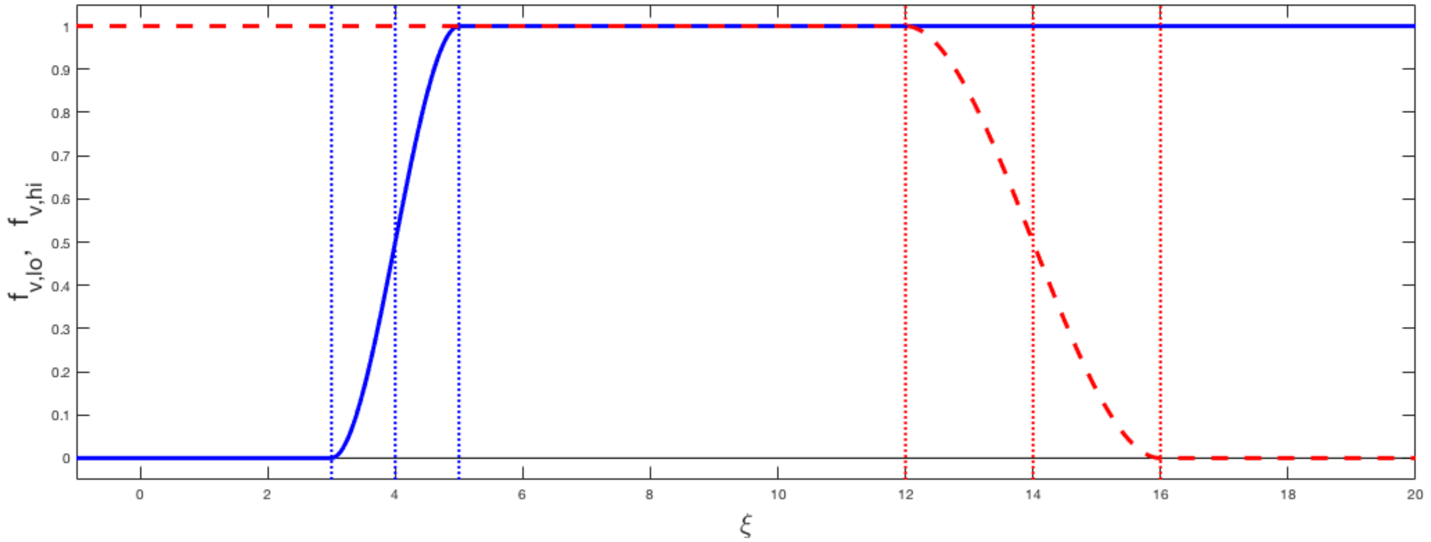

Figure 2. Same as Fig.1. Vertical dotted lines mark the edge locations and the edge widths, in ascending order, as follows:

blue verticals: $\xi_{\mathrm{lo}} - W_{v,\mathrm{lo}}/2 = 3,\quad \xi_{\mathrm{lo}} = 4,\quad \xi_{\mathrm{lo}} + W_{v,\mathrm{lo}}/2 = 5,$

red verticals: $\xi_{\mathrm{hi}} - W_{v,\mathrm{hi}}/2 = 12,\quad \xi_{\mathrm{hi}} = 14,\quad \xi_{\mathrm{hi}} + W_{v,\mathrm{hi}}/2 = 16,$

Copyright © 2020 University of Georgia. Unauthorized duplication or distribution prohibited.

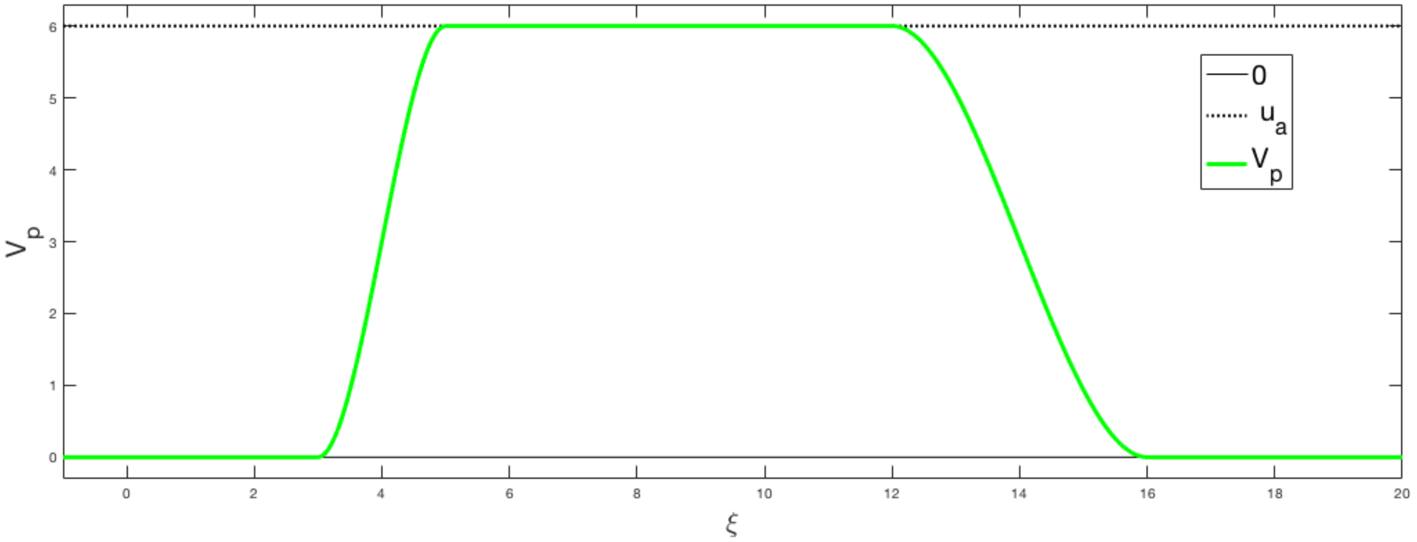

Figure 3. Advection velocity profile shape function,

$$V_{\rm p}(\xi) \;=\; u_{\rm a} \;\; f_{v,{\rm lo}}(\xi) \; f_{v,{\rm hi}}(\xi) \;,$$

plotted *vs.* ξ, for Option 1 Eqs. 45-48, and velocity, edge and width parameters

$$u_{\rm a} \;=\; 6 \;, \quad \xi_{\rm lo} \;=\; 4 \;, \quad W_{v,{\rm lo}} \;=\; 2 \;, \quad \xi_{\rm hi} \;=\; 14 \;, \quad W_{v,{\rm hi}} \;=\; 4 \;.$$

The edge and width parameters are the same as in Figs. 1 and 2.

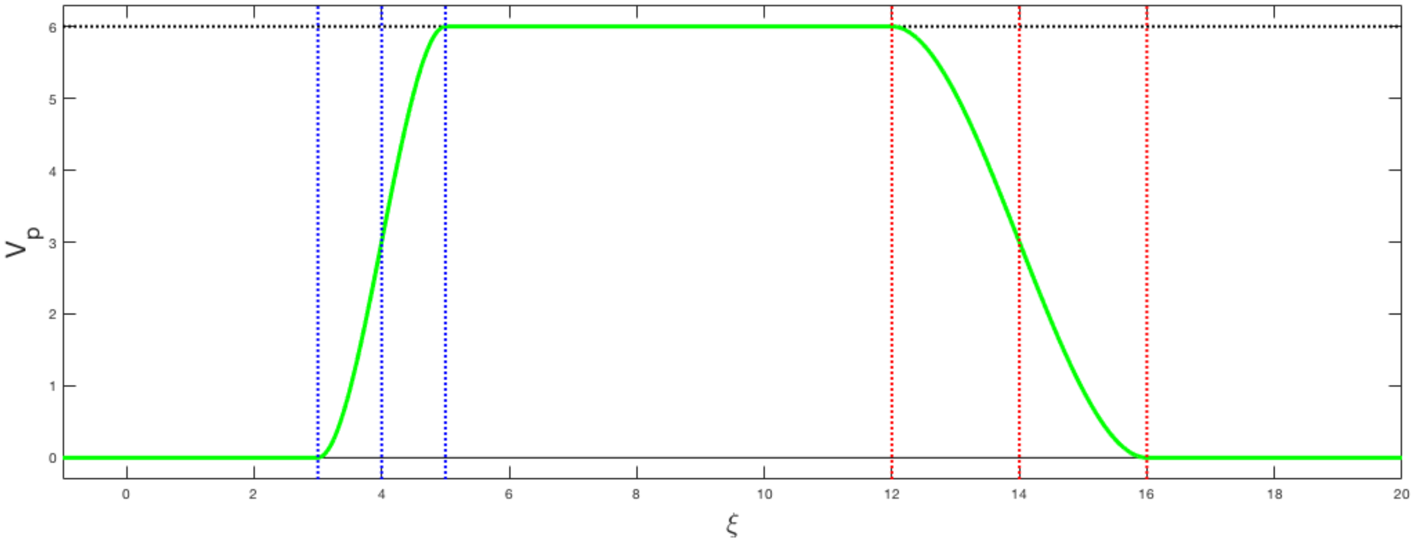

Figure 4. Same as Fig.3. Vertical dotted lines mark the edge locations and the edge widths, in ascending order, as follows:

blue verticals: $\xi_{\rm lo} - W_{v,{\rm lo}}/2 \;=\; 3, \quad \xi_{\rm lo} \;=\; 4, \quad \xi_{\rm lo} + W_{v,{\rm lo}}/2 \;=\; 5,$

red verticals: $\xi_{\rm hi} - W_{v,{\rm hi}}/2 \;=\; 12, \quad \xi_{\rm hi} \;=\; 14, \quad \xi_{\rm hi} + W_{v,{\rm hi}}/2 \;=\; 16,$

Copyright © 2020 University of Georgia. Unauthorized duplication or distribution prohibited.

<u>Advection Velocity Profile Option 2:</u> Algebraic Step Functions

$$f_{v,\mathrm{lo}}(\xi) = q_{\mathrm{a,lo}}\left(\frac{\xi - \xi_{\mathrm{lo}}}{W_{v,\mathrm{lo}}}\right) \quad (52)$$

$$f_{v,\mathrm{hi}}(\xi) = q_{\mathrm{a,hi}}\left(\frac{\xi_{\mathrm{hi}} - \xi}{W_{v,\mathrm{hi}}}\right) \quad . \quad (53)$$

where

$$q_{\mathrm{a,lo}}(\beta) = \frac{1}{2} - \frac{1}{2}\frac{\beta}{\left[\,1 + |\,\beta\,|^{\epsilon_{v,\mathrm{lo}}}\right]^{1/\epsilon_{v,\mathrm{lo}}}} \,, \quad (54)$$

$$q_{\mathrm{a,hi}}(\beta) = \frac{1}{2} - \frac{1}{2}\frac{\beta}{\left[1 + |\,\beta\,|^{\epsilon_{v,\mathrm{hi}}}\right]^{1/\epsilon_{v,\mathrm{hi}}}} \,, \quad (55)$$

the β-variable is defined as in Eq. 48, and the exponents $\epsilon_{v,\mathrm{lo}}$ and $\epsilon_{v,\mathrm{hi}}$ should obey

$$\epsilon_{v,\mathrm{lo}} \geq 1, \quad \text{and} \quad \epsilon_{v,\mathrm{hi}} \geq 1 \,. \quad (56)$$

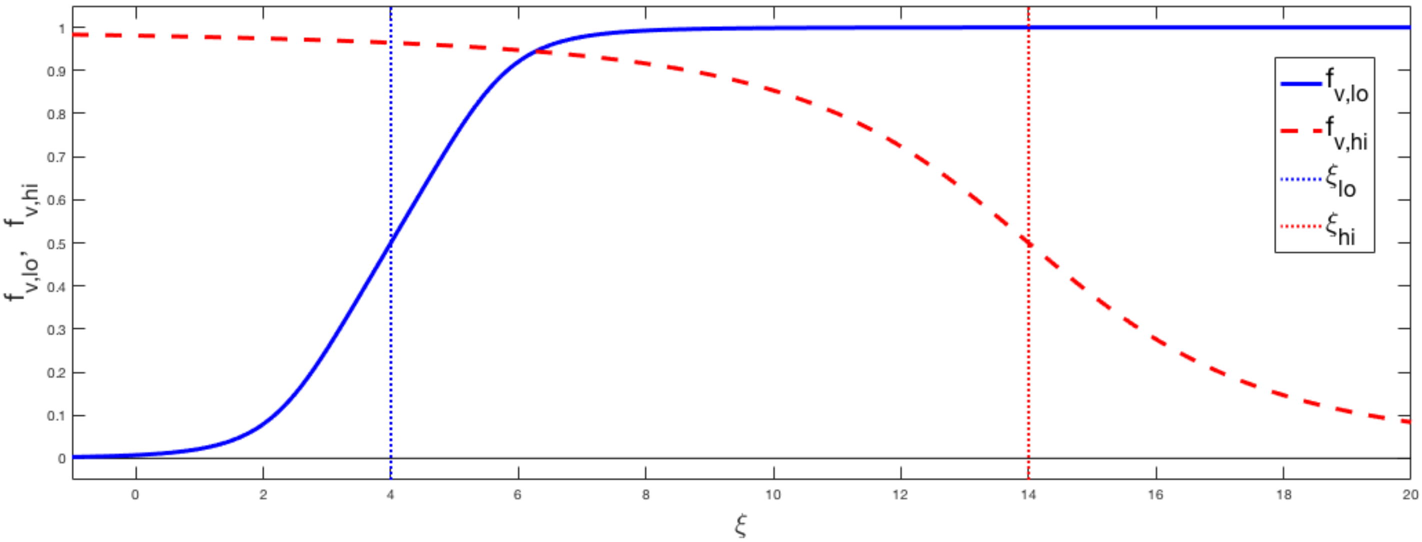

Figure 5. Soft-edge step functions, $f_{v,\mathrm{lo}}(\xi)$ and $f_{v,\mathrm{hi}}(\xi)$, from Option 2 Eqs. 52-55, plotted *vs.* ξ, for edge and width parameters $\xi_{\mathrm{lo}} = 4$, $W_{v,\mathrm{lo}} = 2$, $\epsilon_{v,\mathrm{lo}} = 4$, $\xi_{\mathrm{hi}} = 14$, $W_{v,\mathrm{hi}} = 4$. $\epsilon_{v,\mathrm{hi}} = 2$.

Copyright © 2020 University of Georgia. Unauthorized duplication or distribution prohibited.

Initial Condition (IC) Profile. The PDE system is solved subject to initial conditions, imposed at time $t = 0$ of the form

$$Y_k^{(\mathrm{ini})}(x) \;=\; y_k \; F_{\mathrm{p}}(x) \qquad \text{for species } k = 1, 2, ..., K \;. \tag{57}$$

Here, the y_k denote properly rescaled initial values, mostly derived from the ICs of the single-cell clock model, as described in detail below. The IC profile shape function, $F_{\mathrm{p}}(x)$, determines the spatial variation of the ICs and is assumed to be the same for all species, k. $F_{\mathrm{p}}(x)$ is designed to model an initial inocculation profile in a race tube or in a microdevice channel, with assumed functional form analogous to the $V_{\mathrm{p}}(\xi)$-profile in Eq. 44:

$$F_{\mathrm{p}}(x) \;=\; f_{i,\mathrm{lo}}(x)\; f_{i,\mathrm{hi}}(x) \;. \tag{58}$$

Here, $f_{i,\mathrm{lo}}(x)$ and $f_{i,\mathrm{hi}}(x)$ are, resp., soft-edge upward and downward step functions, defined in *exactly the same way* as the corresponding step functions $f_{v,\mathrm{lo}}(\xi)$ and $f_{v,\mathrm{hi}}(\xi)$ in Eqs. 45-48 (Option 1) and in Eqs. 52-56 (Option 2):

<u>Initial Condition Profile Option 1:</u> Piecewise Cubic Step Functions

$f_{i,\mathrm{lo}}(x)$ is defined by Eq. 45 for $f_{v,\mathrm{lo}}(\xi)$, and $f_{i,\mathrm{hi}}(x)$ is defined by Eq. 46 for $f_{v,\mathrm{hi}}(\xi)$, with following variable name replacements:

$$\begin{aligned}
\xi &\;\rightarrow\; x \;, && (59)\\
\xi_{\mathrm{lo}} &\;\rightarrow\; x_{\mathrm{lo}} \;, &&\\
W_{v,\mathrm{lo}} &\;\rightarrow\; W_{i,\mathrm{lo}} \;, && (60)\\
\xi_{\mathrm{hi}} &\;\rightarrow\; x_{\mathrm{hi}} \;, &&\\
W_{v,\mathrm{hi}} &\;\rightarrow\; W_{i,\mathrm{hi}} \;. && (61)
\end{aligned}$$

<u>Initial Condition Profile Option 2:</u> Algebraic Step Functions

$f_{i,\mathrm{lo}}(x)$ is defined by Eq. 52 for $f_{v,\mathrm{lo}}(\xi)$, and $f_{i,\mathrm{hi}}(x)$ is defined by Eq. 53 for $f_{v,\mathrm{hi}}(\xi)$, with following variable name replacements:

$$\begin{aligned}
\xi &\;\rightarrow\; x \;, && (62)\\
\xi_{\mathrm{lo}} &\;\rightarrow\; x_{\mathrm{lo}} \;, &&\\
W_{v,\mathrm{lo}} &\;\rightarrow\; W_{i,\mathrm{lo}} \;, &&\\
\epsilon_{v,\mathrm{lo}} &\;\rightarrow\; \epsilon_{i,\mathrm{lo}} \;, && (63)\\
\xi_{\mathrm{hi}} &\;\rightarrow\; x_{\mathrm{hi}} \;, &&\\
W_{v,\mathrm{hi}} &\;\rightarrow\; W_{i,\mathrm{hi}} \;, &&\\
\epsilon_{v,\mathrm{hi}} &\;\rightarrow\; \epsilon_{i,\mathrm{hi}} \;. && (64)
\end{aligned}$$

Copyright © 2020 University of Georgia. Unauthorized duplication or distribution prohibited.

Rescaling of User Input Parameters. In the following, let

$$\hat{y} \;:=\; [y_1, ..., y_K] \tag{65}$$

denote the array of all species' initial value factors, y_k, entering into Eq. 57 for the species initial conditions. Also, let

$$\hat{r} \;=\; [r_1, ..., r_M] \tag{66}$$

denote the array of all M model parameters, r_m, that enter into the foregoing PDE system, consisting of Eqs. 24, 26, 28, 30- 35 and 37- 40. Thus, $\hat{r}$ includes, for example, all kinetic rate and transport coeffs, advection velocity parameters, as well as geometrical parameters, such as incocculation and advection zone edge positions and widths.

The code provides complete arrays of default settings for the user input (UI) values for all the foregoing IC and PDE parameters, here to be denoted by $\hat{y}^{(\mathrm{UI})}$ and $\hat{r}^{(\mathrm{UI})}$, resp. The UI parameter values are derived from our earlier modeling work, based on systems of single cells that are not subject to physical transport of cells or nuclei. For that reason, some of these UI values are not suitable to be directly entered into the above PDE system and must be modified by appropriate rescaling transformations. Specifically, the default UI values of the frq and ccg gene concentrations do not satisfy the sum rule Eqs. 18-23. Along with several gene activation-related rate coeffs, r_m, the code therefore rescales the y_k-values of frq and ccg gene and gene activation-related species. This is done in such a way that the overall clock dynamics is not affected by the rescaling, but the sum rules are satisfied, as follows:

$$[frq^0]^{(y)} \;=\; \rho^{(y)} \;\times\; [frq^0]^{(\mathrm{UI})} \;/\; [frq]^{(\mathrm{UI})}_{\mathrm{Tot}} \tag{67}$$

$$[frq^1]^{(y)} \;=\; \rho^{(y)} \;\times\; [frq^1]^{(\mathrm{UI})} \;/\; [frq]^{(\mathrm{UI})}_{\mathrm{Tot}} \tag{68}$$

$$[ccg^0]^{(y)} \;=\; \rho^{(y)} \;\times\; [ccg^0]^{(\mathrm{UI})} \;/\; [ccg]^{(\mathrm{UI})}_{\mathrm{Tot}} \tag{69}$$

$$[ccg^1]^{(y)} \;=\; \rho^{(y)} \;\times\; [ccg^1]^{(\mathrm{UI})} \;/\; [ccg]^{(\mathrm{UI})}_{\mathrm{Tot}} \tag{70}$$

$$[WCC]^{(y)} \;=\; [WCC]^{(\mathrm{UI})} \;/\; [frq]^{(\mathrm{UI})}_{\mathrm{Tot}} \tag{71}$$

$$[WC{-}1]^{(y)} \;=\; [WC{-}1]^{(\mathrm{UI})} \;/\; [frq]^{(\mathrm{UI})}_{\mathrm{Tot}} \tag{72}$$

$$[WC{-}2]^{(y)} \;=\; [WC{-}2]^{(\mathrm{UI})} \;/\; [frq]^{(\mathrm{UI})}_{\mathrm{Tot}} \tag{73}$$

$$[S_3] \;=\; [S_3]^{(\mathrm{UI})} \;\times\; [frq]^{(\mathrm{UI})}_{\mathrm{Tot}} \tag{74}$$

$$[S_4] \;=\; [S_4]^{(\mathrm{UI})} \;\times\; [frq]^{(\mathrm{UI})}_{\mathrm{Tot}} \tag{75}$$

$$[A] \;=\; [A]^{(\mathrm{UI})} \;\times\; \left([frq]^{(\mathrm{UI})}_{\mathrm{Tot}}\right)^n \tag{76}$$

$$[A_c] \;=\; [A_c]^{(\mathrm{UI})} \;\times\; \left([frq]^{(\mathrm{UI})}_{\mathrm{Tot}}\right)^n \tag{77}$$

$$[C_2] \;=\; [C_2]^{(\mathrm{UI})} \;\times [frq]^{(\mathrm{UI})}_{\mathrm{Tot}} \tag{78}$$

$$[C_3] \;=\; [C_3]^{(\mathrm{UI})} \;\times [frq]^{(\mathrm{UI})}_{\mathrm{Tot}} \tag{79}$$

$$[C_4] \;=\; [C_4]^{(\mathrm{UI})} \;\times [frq]^{(\mathrm{UI})}_{\mathrm{Tot}} \tag{80}$$

$$[L_1] \;=\; [L_1]^{(\mathrm{UI})} \;/\; [frq]^{(\mathrm{UI})}_{\mathrm{Tot}} \tag{81}$$

$$[L_2] \;=\; [L_2]^{(\mathrm{UI})} \;/\; [frq]^{(\mathrm{UI})}_{\mathrm{Tot}} \tag{82}$$

$$[S_c] \;=\; [S_c]^{(\mathrm{UI})} \;\times\; [ccg]^{(\mathrm{UI})}_{\mathrm{Tot}} \tag{83}$$

Copyright © 2020 University of Georgia. Unauthorized duplication or distribution prohibited.

where

$$[frq]_{\text{Tot}}^{(\text{UI})} \;=\; [frq^0]^{(\text{UI})} \;+\; [frq^1]^{(\text{UI})} \tag{84}$$

$$[ccg]_{\text{Tot}}^{(\text{UI})} \;=\; [ccg^0]^{(\text{UI})} \;+\; [ccg^1]^{(\text{UI})} \;. \tag{85}$$

The (y)-superscripted concentration values on the LHS of Eqs. 67-73 are then used by the code as the actual y_k-factors, entering into their resp. inital conditions, Eq. 57. So, for example, the IC actually imposed on the PDE solution of species WCC at time t=0 is given by

$$[WCC](x,t)\Big|_{t=0} \;\equiv\; [WCC]^{(\,(\text{ini})}(x) \;=\; [WCC]^{(y)}\; F_{\text{p}}(x)\;. \tag{86}$$

The user-supplied factor $\rho^{(y)}$ in Eqs. 67-70 is the y_k-factor, entering into the inital condition, Eq. 57, for the total nuclear density, such that

$$\rho(x,t)\Big|_{t=0} \;\equiv\; \rho^{(\,(\text{ini})}(x) \;=\; \rho^{(y)}\; F_{\text{p}}(x)\;. \tag{87}$$

From Eqs. 67-70 and Eq. 57, we then see that the ICs of the frq and ccg gene concentrations and the total nuclear density do indeed obey the sum rule Eqs. 22 and 23, since analogous sum rules are obeyed by the rescaled y_k-factors for the gene concentrations, $[frq^0]^{(y)}$, $[frq^1]^{(y)}$, $[ccg^0]^{(y)}$ and $[ccg^1]^{(y)}$:

$$[frq^0]^{(y)} \;+\; [frq^1]^{(y)} \;=\; \rho^{(y)}\;, \tag{88}$$

$$[ccg^0]^{(y)} \;+\; [ccg^1]^{(y)} \;=\; \rho^{(y)}\;. \tag{89}$$

The y_k-factors entering into the IC Eq. 57 for the partial nuclear densities are denoted by $\rho_{00}^{(y)}$, $\rho_{01}^{(y)}$, $\rho_{10}^{(y)}$ and $\rho_{11}^{(y)}$, resp. Their values are set automatically by the code such that the remaining four y_k-sum rules, following from Eqs. 18-21, are satisfied:

$$\rho_{00}^{(y)} \;=\; [frq^0]^{(y)} \;+\; [ccg^0]^{(y)} \;+\; \rho_{11}^{(y)} \;-\; \rho^{(y)} \tag{90}$$

$$\rho_{01}^{(y)} \;=\; \rho^{(y)} \;-\; [ccg^0]^{(y)} \;-\; \rho_{11}^{(y)} \tag{91}$$

$$\rho_{10}^{(y)} \;=\; \rho^{(y)} \;-\; [frq^0]^{(y)} \;-\; \rho_{11}^{(y)}\;. \tag{92}$$

Given $[frq^0]^{(y)}$, $[frq^1]^{(y)}$, $[ccg^0]^{(y)}$, $[ccg^1]^{(y)}$ and $\rho^{(y)}$, the y_k-factor sum rules, derivable from Eqs. 18-23, do not determine a unique value for $\rho_{11}^{(y)}$. However, the positivity requirements for all gene concentrations and and partial nuclear denstities constrain $\rho_{11}^{(y)}$ by

$$\rho_{11,\text{min}}^{(y)} \;\le\; \rho_{11}^{(y)} \;\le\; \rho_{11,\text{max}}^{(y)} \tag{93}$$

where

$$\rho_{11,\text{min}}^{(y)} \;:=\; \max\Big(0,\; [frq^1]^{(y)} \;-\; [ccg^0]^{(y)}\Big)\;, \tag{94}$$

$$\rho_{11,\text{max}}^{(y)} \;:=\; \min\Big([frq^1]^{(y)},\; [ccg^1]^{(y)}\Big) \tag{95}$$

The code then automatically sets

$$\rho_{11}^{(y)} \;=\; \frac{1}{2}\Big(\rho_{11,\text{min}}^{(y)} \;+\; \rho_{11,\text{max}}^{(y)}\Big)\;. \tag{96}$$

Copyright © 2020 University of Georgia. Unauthorized duplication or distribution prohibited.

This arbitrary choice of $\rho_{11}^{(y)}$ affects neither the PDE solutions, $Y_k(x,t)$, for any molecular species, nor the PDE solution for the total nuclear density, $\rho(x,t)$, since the partial nuclear densities do not enter directly into the rate functions of molecular species concentrations or total nuclear density.

For the IC parameters, y_k, of all species and all PDE model parameters, r_m, *not* explicitly specified by the foregoing Eqs. 67-96, the user-supplied input values are used without any modifications as the inputs into the IC settings and into the PDE model equations. In other words, in the IC Eq. 57, the code sets

$$y_k \; = \; y_k^{(\mathrm{UI})} \tag{97}$$

for the IC y_k-parameter values of all species, k, not explicitly included in Eqs. 67-73, 88, 89, or 90 - 96. Likewise, in the PDE system defined by Eqs. 24, 26, 28, 30- 35 and 37- 40, the code sets

$$r_m \; = \; r_m^{(\mathrm{UI})} \tag{98}$$

for all PDE r_m-parameter values not explicitly specified by Eqs. 74-83

The purpose of the foregoing UI rescaling procedure is to allow the user to choose *any* combination of UI values for the gene concentration and nuclear density IC parameters, $[frq^0]^{(\mathrm{UI})}$, $[frq^1]^{(\mathrm{UI})}$, $[ccg^0]^{(\mathrm{UI})}$, $[ccg^1]^{(\mathrm{UI})}$, and $\rho^{(y)}$, without regard to any sum rule compliance. The automatic IC and PDE model input parameter settings performed by the code, using Eqs. 67-85 and Eqs. 90-96, then ensures that the nuclear density IC is indeed determined by the user-supplied $\rho^{(y)}$-value and that the PDE solution obeys all required sum rules, when all dynmaical variables, $Y_{\mathrm{k}}(x,t)$, are initialized by the IC Eq. 57. The user-supplied input values for $[frq^0]^{(\mathrm{UI})}$, $[frq^1]^{(\mathrm{UI})}$, $[ccg^0]^{(\mathrm{UI})}$ and $[ccg^1]^{(\mathrm{UI})}$ then determine only the *ratios* of the various gene concentrations, but not their absolute values, since, by Eqs. 67-70,

$$\frac{[frq^1]^{(y)}}{[frq^0]^{(y)}} \; = \; \frac{[frq^1]^{(\mathrm{UI})}}{[frq^0]^{(\mathrm{UI})}} \qquad \text{and} \qquad \frac{[ccg^1]^{(y)}}{[ccg^0]^{(y)}} \; = \; \frac{[ccg^1]^{(\mathrm{UI})}}{[ccg^0]^{(\mathrm{UI})}} \; . \tag{99}$$

Space-Time Grid. The PDE system is approximated by an ordinary differential equation (ODE) system, by way of discretizing the x-domain, with an x-Grid defined by

$$x_j \; = \; dx\,(j-1) \qquad \text{for } j = 1,2,...,N_{\mathrm{L}}. \tag{100}$$

where N_{L} is the number of x-grid points and and dx is the x-grid spacing:

$$dx \; = \; L/(N_{\mathrm{L}}-1) \tag{101}$$

The resulting ODE system is solved numerically and the solution, $Y_k(x_j,t_\ell)$, for selected species, k, is written out and/or plotted for all x-grid points, x_j, and for a grid of output times, t_ℓ, defined by:

$$t_\ell \; = \; dt\,(\ell-1) \qquad \text{for} \quad \ell = 1,2,...,(N_{\mathrm{T}}+1). \tag{102}$$

where $N_{\mathrm{T}}+1$ is the number of output t-grid points, T is the length of the PDE solution time interval, and dt is the t-grid spacing:

$$dt \; = \; T/N_{\mathrm{T}} \; . \tag{103}$$

Copyright © 2020 University of Georgia. Unauthorized duplication or distribution prohibited.

B. Numerics Control and Model Input Parameters

This section provides a (nearly) complete tabulation and variable name translation dictionary for all input parameter variables for the clock tube code that must be supplied by the user. In each subsection below, a dictionary list of user-supplied input parameter variables appears under the headline

User Input Variables:

In addition, just for clarity and background information, there are also a few dictionary lists of dependent variables which the code automatically calculates from the user-supplied inputs. Each such list of dependent variables appears under the headline

Code Derived Variables and Arrays:

The user *should not change* any of the code statements relating to these dependent variables, unless (s)he is absolutely sure (s)he knows what (s)he's doing!!

For PDE model UI parameters included in $\hat{r}$, such as kinetic rate and transport coeffs, the in-code variable names of these input parameters are stored in a cell array named "rPm.PkinName". Each user-supplied entry of such a UI parameter is thus immediately followed by a line for the corresponding variable name storage operation. For example, for the UI entry of the L_2-parameter, this pair of Matlab statement lines might look like this:

```
rk_Ori.L2= 14.896;
   iPkin=iPkin+1; rPm.PkinName{iPkin} ='rk.L2';
```

... and for the UI entry of the S_4-parameter, it might look like this:

```
rk_Ori.S4= 7.67992;
   iPkin=iPkin+1; rPm.PkinName{iPkin} ='rk.S4';
```

Again, the user *should not change* this pairwise sequence of UI parameter entry and variable name storage statements, nor any other code statements relating to this cell array, "rPm.PkinName", unless (s)he is absolutely sure (s)he knows what (s)he's doing!!

This information about the cell array "rPm.PkinName" is provided here only for more advanced users who want the make upgrades to the code and, in the process, introduce new $\hat{r}$-parameter UI variables, related to kinetic rate or transport coefficients, *etc.* of the PDE model. Any such new input $\hat{r}$-parameter variables should be entered into the "rPm.PkinName" variable name list, in exactly the format shown above, as also found inside the Matlab source code file, called *Tube_... .m.*.

The user *should also ignore and make no changes* to any (apparent) input variables found in the code which are not listed below or not documented in Section A. above.

For most of the user-suplied input parameters, the input values must be entered by the user inside the source code fct named

SetPmKI.

For a small subset of the user-suplied input parameters, which control the output of results

Copyright © 2020 University of Georgia. Unauthorized duplication or distribution prohibited.

(in figures and data files), the input values must be set by the user, inside the source code, in the main fct named

Tube.

In each sub-section below, the name of this input function is stated in parentheses, as either ***SetPmKI*** or ***Tube***, next to the sub-section header.

Space-Time Grid (*SetPmKI*).

User Input Variables:

$$N_{\mathrm{L}} \;==\; \mathrm{rPm.nCells} \tag{104}$$
$$L \;==\; \mathrm{rPm.xLength} \tag{105}$$

$$N_{\mathrm{T}} \;==\; \mathrm{rPm.nTimeSol} \tag{106}$$
$$T \;==\; \mathrm{rPm.tfinSol} \tag{107}$$

Code Derived Variables and Arrays:

$$dx \;==\; \mathrm{rPm.dx} \tag{108}$$
$$x_j \;==\; \mathrm{rPm.xGr} \tag{109}$$

$$dt \;==\; \mathrm{rPm.dt} \tag{110}$$
$$t_\ell \;==\; \mathrm{rPm.tGr} \tag{111}$$

Species IC y_k-Factors and Species Names (*SetPmKI*).

User Input Variables:

$$\{\; y_k^{(\mathrm{UI})},\; \mathrm{NameSpec}_k \;\} \;==\; \mathrm{Spec\_Ori} \tag{112}$$

"Spec_Ori" is a $K \times 2$ cell array. It stores the un-rescaled (UI) initial value factors, $y_k^{(\mathrm{UI})}$, as a numerical value, in the 1st column and the conventional species name, as a character string, in the 2nd column. The user should *change only* the $y_k^{(\mathrm{UI})}$-value, if needed; the user *must not change* the species name, "Namespec$_k$", in "Spec_Ori".

Ignore and do not change the input cell array "Spec_Ori_XG". Ignore and do not change the input cell arrays "DpenName" and "DpenName_XG".

The only y_k-value not to be entered in the "Spec_Ori" cell array is the y_k-value for the total nuclear density:

$$\rho^{(y)} \;==\; \mathrm{rk\_Ori.RhoIMod} \tag{113}$$

Copyright © 2020 University of Georgia. Unauthorized duplication or distribution prohibited.

Standard Intra-Cell Rate Coeffs (*SetPmKI*).

User Input Variables:

$$j_{NonCatA} \;==\; \text{rPm.rk.jNonCatA} \tag{114}$$
$$= 0\text{: } A\text{-}\bar{A} \text{ reaction is catalytic. Keep } = 0!$$
$$= 1\text{: } A\text{-}\bar{A} \text{ reaction is non-catalytic}$$
$$n \;==\; \text{rPm.rk.nCoopA} \tag{115}$$
$$m \;==\; \text{rPm.rk.nCoopP} \tag{116}$$
$$A \;==\; \text{rk\_Ori.A} \tag{117}$$
$$\bar{A} \;==\; \text{rk\_Ori.Abar} \tag{118}$$
$$\text{... etc. ...}$$
$$S_4 \;==\; \text{rk\_Ori.S4} \tag{119}$$
$$S_{\rm c} \;==\; \text{rk\_Ori.Sc} \tag{120}$$

The in-code variable names for standard intra-cell rate coeff parameters, Eqs. 115-120, are self-explanatory: they follow the naming conventions set forth in our standard diagram for the clock network model with an internal signal, Si, see figure attached below. The foregoing tabulation therefore shows only a small subset of these variable names, listed in their in-code order, with "... etc. ..." indicating all other standard UI parameters not explicitly shown here.

Intra- and Extra-Cell Signaling and Signal Diffusion (*SetPmKI*).

User Input Variables:

$$D_{10}/\eta \;==\; \text{rk\_Ori.RatD10Eta} \tag{121}$$
$$k_{\rm ICM} \equiv \eta \;==\; \text{rk\_Ori.Eta} \tag{122}$$
$$D_9 \;==\; \text{rk\_Ori.D9} \tag{123}$$
$$C_4 \;==\; \text{rk\_Ori.C4} \tag{124}$$
$$k_{Si} \;==\; \text{rk\_Ori.kSi} \tag{125}$$
$$\mathcal{V}_{\rm int}/\mathcal{V}_{\rm ext} \;==\; \text{rk\_Ori.Rix} \tag{126}$$
$$\Delta_{Si} \;==\; \text{rk\_Ori.DiffSi} \tag{127}$$
$$\Delta_{Si} \;==\; \text{rk\_Ori.DiffSe} \tag{128}$$

Code Derived Variables and Arrays:

$$D_{10} \;==\; \text{rk\_Ori.D10} \tag{129}$$
$$k_{\rm XCM} \;==\; \text{rk\_Ori.EtaRix} \tag{130}$$

Copyright © 2020 University of Georgia. Unauthorized duplication or distribution prohibited.

Nuclear Proliferation and Nutrient Consumption (*SetPmKI*).

User Input Variables:

$$k_{\rm ND} \;==\; \text{rk\_Ori.kDivN} \tag{131}$$
$$\rho^{(y)} \;==\; \text{rk\_Ori.RhoIMod} \tag{132}$$
$$\rho_{\rm Lim} \;==\; \text{rk\_Ori.RhoLim} \tag{133}$$
$$\epsilon_\rho \;==\; \text{rk\_Ori.eRhoLim} \tag{134}$$
$$k_{\rm FC}/k_{\rm ND} \;==\; \text{rk\_Ori.kPhi} \tag{135}$$
$$\tag{136}$$

Light Entrainment (*SetPmKI*).

User Input Variables:

$$j_{Phot} \;==\; \text{rk\_Ori.jPhot} \tag{137}$$
$$\text{... etc. ...}$$
$$e_{Phot} \;==\; \text{rk\_Ori.Phot\_exp} \tag{138}$$

The foregoing variable dictionary list is presently incomplete (... etc. ...), since the interaction of the clock with an external light stimulus is not yet fully documented in Section A. and also not yet fully implemented in the code.

Code Derived Variables and Arrays:

$$\Omega_{Phot} \;==\; \text{rk\_Ori.Phot\_Omega} \tag{139}$$
$$\varphi_{Phot} \;==\; \text{rk\_Ori.Phot\_phShift} \tag{140}$$

Advection Velocity Profile and Transport Control (*SetPmKI*).

User Input Variables:

$$j_{\rm vProfile} \;==\; \text{rPm.vP.jvProfile} \tag{141}$$
$$= 1\text{: Advection velocity profile Option 1}$$
$$= 1\text{: Advection velocity profile Option 2}$$
$$j_{\rm AdvCur} \;==\; \text{rPm.vP.jCurAd} \tag{142}$$
$$\text{Set} = 1\text{. Don't ask!}$$
$$u_{\rm p} \;==\; \text{rPm.vP.uPGro} \tag{143}$$
$$u_{\rm a}/u_{\rm p} \;==\; \text{rPm.vP.fuAdvec} \tag{144}$$
$$\xi_{\rm hi} \;==\; \text{rPm.vP.xVzoneHi0} \tag{145}$$

Copyright © 2020 University of Georgia. Unauthorized duplication or distribution prohibited.

$$\xi_{\text{lo}} \;==\; \text{rPm.vP.xVzoneLo0} \tag{146}$$
$$W_{v,\text{lo}} \;==\; \text{rPm.vP.wEdgVzoneHi} \tag{147}$$
$$W_{v,\text{lo}} \;==\; \text{rPm.vP.wEdgVzoneLo} \tag{148}$$
$$\epsilon_{v,\text{hi}} \;==\; \text{rPm.vP.eVzoneHi} \tag{149}$$
$$\epsilon_{v,\text{lo}} \;==\; \text{rPm.vP.eVzoneLo} \tag{150}$$
$$j_{Si,\text{Diff}} \;==\; \text{rPm.vP.jSiDiff} \tag{151}$$
$$= 0/1\text{: Si diffusion term } (\Delta_{Si}) \text{ is OFF/ON}$$
$$j_{Se,\text{Diff}} \;==\; \text{rPm.vP.jSeDiff} \tag{152}$$
$$= 0/1\text{: Se diffusion term } (\Delta_{Se}) \text{ is OFF/ON}$$
$$j_{Se,\text{Advec}} \;==\; \text{rPm.vP.jSeAdvec} \tag{153}$$
$$= 0/1\text{: Se advection term OFF/ON. Keep} = 0!$$
$$j_{\phi,\text{Advec}} \;==\; \text{rPm.vP.jPhiAdvec} \tag{154}$$
$$= 0/1\text{: } \phi \text{ advection term OFF/ON. Keep} = 0!$$

Initial Condition Profile (*SetPmKI*).

User Input Variables:

$$j_{\text{iProfile}} \;==\; \text{rPm.iP.jiProfile} \tag{155}$$
$$= 1\text{: Initial condition profile Option 1}$$
$$= 2\text{: Initial condition profile Option 2}$$
$$x_{\text{hi}} \;==\; \text{rPm.iP.xIzoneHi0} \tag{156}$$
$$x_{\text{lo}} \;==\; \text{rPm.iP.xIzoneLo0} \tag{157}$$
$$W_{i,\text{lo}} \;==\; \text{rPm.iP.wEdgIzoneHi} \tag{158}$$
$$W_{i,\text{lo}} \;==\; \text{rPm.iP.wEdgIzoneLo} \tag{159}$$
$$\epsilon_{i,\text{hi}} \;==\; \text{rPm.iP.eIzoneHi} \tag{160}$$
$$\epsilon_{i,\text{lo}} \;==\; \text{rPm.iP.eIzoneLo} \tag{161}$$
$$\tag{162}$$

Copyright © 2020 University of Georgia. Unauthorized duplication or distribution prohibited.
