# Supplementary material for: The clock in growing hyphae and their synchronization in Neurospora crassa
Source: Commun Biol. 2024 Jun 18;7:735. doi: 10.1038/s42003-024-06429-6 (PMC11189396; doi:10.1038/s42003-024-06429-6)
Supplement: Supplementary file 8 — Supplementary Data 3 [file 42003_2024_6429_MOESM8_ESM.pdf]

Supplement to Filament Clock Model: The Partial Differential Equation System

A. Model Equations and Dynamical Variables

The Filament Clock Model is a partial differential equation (PDE) system, describing the spatio-temporal evolution of a multi-nuclear clock system that is undergoing 1D spatial growth of its filamental cellular domain. The PDE system model may be applicable to the 1D growth of a *N. crassa* cell population into a race tube, or to the growth of *N. crassa* fungal hyphae into microdevice channels. The growth of the intra-cellular hyphal filament volume is driven by an uptake of liquid matter from the extra-cellular space, near the hyphal growth front, accompanied by the advection (drift) of intra-cellular matter towards the hyphal growth front.

Dynamical Variables. Molecular species concentrations and other space-time-dependent dynamical variables of the PDE model are denoted by $Y_k(x,t)$ where k is an integer index, t is time and x is the spatial coordinate, defined on an x-domain of length L, with

$$0 \;\leq\; x \;\leq\; L \; . \tag{1}$$

The PDE system for these dynamcical variables, described below, is solved on a finite time domain, of duration T, such that

$$0 \;\leq\; t \;\leq\; T \; . \tag{2}$$

At time $t = 0$, user-supplied initial conditions (ICs), $Y_k^{(\text{ini})}(x)$, are imposed, for all k and x, by setting

$$Y_k(x,0) \;=\; Y_k^{(\text{ini})}(x) \; . \tag{3}$$

The index $k = 1, 2, ...K$ is used internally in the PDE solution code to enumerate the K dynamical variables, $Y_k(x,t)$, evolving in time and space according to our model PDE system. These variables include $K - 6$ intracellular molecular species, also shown in the network diagram figure in the main article, with the intra-cellular signal, Si, enumerated as $[Si](x,t) \equiv Y_{K–6}(x,t)$. All intra-cellular species, $k = 1, ..., (K-6)$, are identified with their respective PDE rate functions in Section B. The remaining six dynamical variables, *i.e.*, $Y_k(x,t)$ with $k = K-5, K-4, ..., K$, are explained in the following and also identified with their respective PDE rate functions in Section B.

In addition to the K–6 intra-cell molecular species, we have to introduce seven new dynamical variables into the model in order to describe the effects of extra-cell signaling, mitotic nuclear division, and its dependence on an ambient nutrient supply. Specifically, we need to keep track of how nuclear division processes affect (sub-)populations of nuclei with different gene activation states. To that end, we denote the four possible activation states of the (frq, ccg) gene pair in each nucleus by

$$00 \;=\; frq = \text{OFF}, \quad ccg = \text{OFF} \tag{4}$$

$$01 \;=\; frq = \text{OFF}, \quad ccg = \text{ON} \tag{5}$$

$$10 \;=\; frq = \text{ON}, \qquad ccg = \text{OFF} \tag{6}$$
$$11 \;=\; frq = \text{ON}, \qquad ccg = \text{ON} \tag{7}$$

The state "ON" indicates a frq-gene or a ccg whose WCC-activator binding site is occupied by the full complement of $n_{\rm W}$ WCC-molecules; "OFF" indicates the binding site to be unoccupied. Here $n_{\rm W}$ denotes the cooperativity exponent of the gene activation. That is, $n_{\rm W}$ is number of WCC-molecules required to occupy the binding sites for the respective gene to be activated.

We can then partition the population of all nuclei into four "gene-state resolved" sub-populations, based on the foregoing four activation states, with partial densities of these four sub-populations denoted, respectively, by

$$\rho_{00}(x,t)\ , \quad \rho_{01}(x,t)\ , \quad \rho_{10}(x,t)\ , \quad \rho_{11}(x,t)\ . \tag{8}$$

The total nuclear density, denoted by $\rho(x,t)$, is then given by

$$\rho(x,t) \;=\; \rho_{00}(x,t) \;+\; \rho_{01}(x,t) \;+\; \rho_{10}(x,t) \;+\; \rho_{11}(x,t)\ . \tag{9}$$

In addition, we want to keep track of, potentially, two extra-cellular species: the concentrations of the extra-cellular signal, $[Se](x,t)$, and an extra-cellular nutrient concentration, denoted by $\Phi(x,t)$. The latter can be used to model possible effects of local nutrient availability and nutrient exhaustion on the mitotic growth of the local nuclear population.

In our enumeration of dynamical variables, $Y_k(x,t)$, the foregoing six species are then included as follows:

$$\rho_{00}(x,t) \;\equiv\; Y_{K\text{–}5}(x,t) \tag{10}$$
$$\rho_{01}(x,t) \;\equiv\; Y_{K\text{–}4}(x,t) \tag{11}$$
$$\rho_{10}(x,t) \;\equiv\; Y_{K\text{–}3}(x,t) \tag{12}$$
$$\rho_{11}(x,t) \;\equiv\; Y_{K\text{–}2}(x,t) \tag{13}$$
$$[Se](x,t) \;\equiv\; Y_{K\text{–}1}(x,t) \tag{14}$$
$$\Phi(x,t) \;\equiv\; Y_{K}(x,t) \tag{15}$$

For the specific model implemented in the code, the complete listing of all PDE dynamical variables by species name is given in Section B: there are $K-6 = 11$ intra-cell molecular species, 4 intra-cell nuclear species, 2 extra-cell species, and hence

$$K \;=\; 17\ . \tag{16}$$

The total and partial nuclear densities are then related to the densities of the gene states frq^0, frq^1, ccg^0, ccg^1 $wc\text{–}1^1$ and $wc\text{–}2^1$ by the following "sum rules":

$$[frq^0](x,t) \;=\; \rho_{00}(x,t) \;+\; \rho_{01}(x,t) \tag{17}$$
$$[frq^1](x,t) \;=\; \rho_{10}(x,t) \;+\; \rho_{11}(x,t) \tag{18}$$

$$
\begin{aligned}
[ccg^0](x,t) &= \rho_{00}(x,t) \;+\; \rho_{10}(x,t) &(19)\\
[ccg^1](x,t) &= \rho_{01}(x,t) \;+\; \rho_{11}(x,t) &(20)\\
[wc\text{–}1^1](x,t) &= \rho(x,t) &(21)\\
[wc\text{–}2^1](x,t) &= \rho(x,t) &(22)\\
\rho(x,t) &= [frq^0](x,t) \;+\; [frq^1](x,t) &(23)\\
\rho(x,t) &= [ccg^0](x,t) \;+\; [ccg^1](x,t)\;. &(24)
\end{aligned}
$$

In Eqs. 17-22, we are assuming that each nucleus contains only one copy of each of the four genes, frq, ccg, wc–1 and wc–2. If multiple copies of a gene were present in the nucleus, the right-hand side of the sum rule equation for the respective gene states would have to be multiplied by the that gene's copy number.

It is sufficient to include only the partial nuclear densities, $\rho_{00}(x,t)$, $\rho_{01}(x,t)$, $\rho_{10}(x,t)$ and $\rho_{11}(x,t)$, as dynamical variables in the PDE system to be solved, but not the total nuclear density or the frq-, ccg-, wc–1- or wc–2-gene state densities. Instead, we use the foregoing Eqs. 17-22 to calculate the gene state densities, $[frq^0](x,t)$, $[frq^1](x,t)$, $[ccg^0](x,t)$, $[ccg^1](x,t)$, $[wc\text{–}1^1](x,t)$ and $[wc\text{–}2^1](x,t)$, as well as Eq. 9 to calculate the total nuclear density, $\rho(x,t)$, respectively, from the partial nuclear densities. These six gene state densities and the total nuclear density then enter into the calculations of the rate functions for several dynamical variables in the PDE system, specifically for the mRNA concentrations, as detailed below.

The wc–1 and wc–2 genes are assumed to be constitutively in their ON states, $wc\text{–}1^1$ and $wc\text{–}2^1$. Hence, the respective gene state densities $[wc\text{–}1^1](x,t)$ and $[wc\text{–}2^1](x,t)$ are both set equal to the total nuclear density, $\rho(x,t)$, in Eqs. 21-22.

Partial Differential Equation System. The coupled PDE system for the dynamical variables $Y_k(x,t)$ of all K intra-cellular and extra-cellular molecular species and nuclear species has the general form

$$
\partial_t\, Y_k(x,t) \;=\; R_k(\hat{Y}(x,t),t) \;-\; \partial_x \left(r_k\, v(x,t)\, Y_k(x,t)\right) \;-\; \Delta_k\, \partial_x^2 Y_k(x,t) \qquad \text{for} \;\; k = 1, 2, ..., K \qquad (25)
$$

where $\hat{Y}(x,t)$ is short-hand for

$$
\hat{Y}(x,t) \;:=\; [Y_1(x,t),\; ...,\; Y_K(x,t)]\;. \qquad (26)
$$

Here, ∂_t denotes the derivative with respect to time, t; ∂_x and ∂_x^2 denote, respectively, the first and second derivative with respect to position, x.

The rate function $R_k(\hat{Y},t)$ denotes rate of change of the molecular species concentration or partial nuclear density, $Y_k(x,t)$, due to physico-chemical or biological processes which produce or consume species number k. By definition, each $R_k(\hat{Y}(x,t),t)$ comprises all rate contributions which depend only on concentration values, $\hat{Y}(x,t)$, at location x and time t, but do *not* depend on the x-derivatives of any $Y_k(x,t)$. This is described in detail in Section B below.

Note that R_k can acquire an explicit time dependence, *i.e.*, for certain species, k, R_k is a fct of both $\hat{Y}$ *and* of time t, if the clock system is subjected to time-dependent external

perturbations or stimuli, such as a time-varying exposure to light. Mitotic nuclear division processes also contribute to the rate functions, R_k, of the intra-cell partial nuclear densities. In the present work, explicitly time-dependent rate function modifications, *e.g.*, due to light exposure, were not used and are therefore not described or modeled here in any further detail. Nuclear division contributions to the partial nuclear densities are also described in detail in Section B.

The term involving the velocity profile, denoted by $v(x,t)$ in Eq. 25, describes the advection, also referred to as drift, of intra-cellular particles, specifically molecules and nuclei, towards the filamental growth front, due to the flow of intra-cellular liquid matter inside the filament towards the filamental growth tip. The velocity, $v(x,t)$, is defined as the velocity at which the intra-cellular liquid matter is flowing inside the filament, towards the growth tip. This intra-filamental flow is driven by the influx of extra-cellular matter into the filament, from the extra-cellular space.

The influx of extra-cellular matter, combined with fluid flow towards the growing tip, is required, on simple physical grounds, in order to fill up the expanding intra-cellular volume that is continually being created by the growth of the filament tip. In the absence of this influx and resultant flow towards the growing tip, any growth of intra-filamental volume would be prohibited, due to the pressure forces exerted on the filament by the extra-cellular medium, combined with by the incompressibilty or, equivalently, by the inexpandability of the intra-cellular medium.

The flow of the liquid medium inside the filament sweeps along intra-cellular molecules and nuclei that are dissolved or suspended, respectively, in the liquid medium. The resulting flow transport of molecules and nuclei contributes to the rate of change of their respective concentrations and densities. This "advective" contribution to the rate of change of concentrations and densities must be taken into account in addition to the spatially stationary chemical and biological processes which create and/or consume the respective particle species. The latter, reactive rate contributions are modeled by the rate functions R_k in Eq. 25, described in detail in Section B, below. The former, advective rate contributions are mathematically described in terms of advective current densities which we will now describe.

For any given intra-cellular species, k, with concentration $Y_k(x,t)$, being transported along the filament with drift velocity $v_k(x,t)$, the advective concentration current density of the flow of k-particles at location x and time t is given by[1]

$$J_k^{(\mathrm{Adv})}(x,t) \;=\; v_k(x,t)\, Y_k(x,t) \tag{27}$$

$J_k^{(\mathrm{Adv})}(x,t)$ is the rate of particles, per filament cross-sectional area, passing location x at time t with drift velocity $v_k(x,t)$. The negative of the partial spatial derivative of $J_k^{(\mathrm{Adv})}(x,t)$ then gives the advective contribution to the rate of change in the concentration at location x and time t, *i.e.*,

$$\partial_t Y_k(x,t)|_{\mathrm{Adv}} = -\partial_x J_k^{(\mathrm{Adv})}(x,t) \ . \tag{28}$$

This contribution to the rate of change in the concentration is caused by the imbalance between arrival and departure of particles at location x when $J_k^{(\mathrm{Adv})}(x,t)$ is non-uniform in

space. That, in essence, is the origin of the advective rate term in Eq. 25, for each intra-cellular species $k = 1, ..., K-2$. Upon inserting Eq. 27 into Eq. 28, $\partial_t Y_k(x,t)|_{\mathrm{Adv}}$ becomes the advection term shown in Eq. 25.

To model the drift velocites, $v_k(x,t)$, we assume that each $v_k(x,t)$ is propprtional to the flow velocity, by setting

$$v_k(x,t) = r_k\, v(x,t) \tag{29}$$

with a dimensionless proportionality pre-factor r_k, as shown in the advection rate term in Eq. 25. On account of the molecular sizes, it is reasonable to assume that molecular species drift at the same velocities, $v_k(x,t)$, as the molecular components of the intra-celluar liquid medium itself. That is, $v_k(x,t)$ is the same as the flow velocity, $v(x,t)$, of the intra-celluar liquid medium. We therefore set the pre-factors

$$r_k = 1 \qquad \text{for } k = 1, 2, ..., K-6, \tag{30}$$

i.e., for all intra-cellular molecular species in Eqs. 25.

From our microfluids-based observations, we know that the drift velocity of the nuclei is noticeably reduced relative to the intra-celluar flow velocity. This is likely due to the much larger (macroscopic) size of the nuclei which subjects the nuclei to obstructions inside the filament. For example, entanglements and attachments with cyto-skeletal components or intra-filamental septa could cause the nuclear drift to slow down, relative to the flow velocity of intra-cellular liquid medium. In our PDE model, we have therefore set

$$r_k \;=\; r^{(\mathrm{N})} \;<\; 1 \qquad \text{for } k = K-5,\; K-4,\; K-3,\; K-2, \tag{31}$$

i.e., for the drift motion of all nuclear species concentrations, $Y_k(x,t)$, as defined in Eqs. 10-13. The $r^{(\mathrm{N})}$-value tabulated in Section D was used for all results shown in this article. It is based on our microfluidics measurements of the nuclear drift velocity and of the filamental tip growth velocity.

Lastly, the two extra-celluar species in our PDE system, $k = K-1$ and K, are not subject to advection by the intra-cellular fluid flow. We therefore set

$$r_k \;=\; 0 \qquad \text{for } k = K-1,\; K. \tag{32}$$

The last term in Eq. 25, involving the second spatial derivative describes the rate of change of conctration effected by diffusion transport of the respectve species, with a diffusion coefficient denoted by Δ_k. In the PDE model results, we include this term only for the extra-cellular signaling species, Se, with $k = K-1$. We therefore set

$$\Delta_k \;=\; \delta_{k,(K-1)}\, \Delta^{(Se)} \qquad \text{for } k = 1,\; ...,\; K. \tag{33}$$

where $\delta_{k,k'} = 1$ if $k = k'$, else $\delta_{k,k'} = 0$, and $\Delta^{(Se)}$ denotes the diffusion coefficient of the signaling species, Se, in the extra-cellular medium. The diffusion rate term in Eq. 25 can be traced back to a concentration current flow that is driven by a spatial gradient of the

respective species concentration itself, *i.e.*, by $\partial_x Y_k(x,t)$. The resulting diffusion current density is then given by Fick's law[2], as

$$J_k^{(\mathrm{Dif})}(x,t) \;=\; -\Delta_k\, \partial_x Y_k(x,t) \;. \tag{34}$$

As in the case of the advection current, any spatial non-uniformity of $J_k^{(\mathrm{Dif})}(x,t)$ changes the concentration of species ks at a rate analogous to Eq. 28, given by:

$$\partial_t Y_k(x,t)|_{\mathrm{Dif}} = -\partial_x J_k^{(\mathrm{Dif})}(x,t) \;. \tag{35}$$

Combined with Eq. 34, $\partial_t Y_k(x,t)|_{\mathrm{Dif}}$ becomes the diffusion term shown in Eq. 25.

Advection Velocity Profile. The flow velocity profile, $v(x,t)$, must be provided as a user-supplied input into the model, as described in detail below. In our model velocity profile, $v(x,t)$ is nonzero only within a finite x-interval, located in immediate proximity to the growth tip and referred to as the advection zone. As a function of time, the advection zone is moving along with the filamental growth tip, at a velocity set equal to the observed filamental growth velocity. The maximal flow velocity inside the advection zone, $u_a \equiv \max(v(x,t))$, is set equal to the filamental tip growth velocity, as dictated by the physics of incompressible flow explained above. That is, the flow velocity in immediate proximity to the growth tip must match the velocity at which the filamental volume is expanding due to the growth.

In detail, the velocity profile, $v(x,t)$, is assumed to have a fixed pulse shape, as a function of position. The pulse is traveling along the x-axis in $(+x)$-direction and is thereby tracking the advancement of the growth tip of the filament. The velocity pulse travel velocity, u_p, must then also match the growth velocity of the advancing growth tip, a condition again strictly enforced by the basic physics of incompressible flow discussed above. For simplicity, we assume u_p to be a constant, *i.e.*, independent of time.

The fixed-shape velocity pulse is modeled by a time-independent shape function $V_\mathrm{p}(\xi)$ where ξ is the position coordinate in the co-moving reference frame of the pulse. $V_\mathrm{p}(\xi)$ is assumed to have a finite support interval, $[\xi_\mathrm{q}, \xi_\mathrm{p}]$, defining the advection zone. That is, $V_\mathrm{p}(\xi) \neq 0$ *iff* $\xi_\mathrm{q} < \xi < \xi_\mathrm{p}$. The construction and shape of $V_\mathrm{p}(\xi)$ are described below and illustrated in Figs.S1-S4. Positions ξ_q and ξ_p mark the rear and the front end of the advection zone on the ξ-axis, indicated by the leftmost blue line and the rightmost red line in S4, respectively.

The time dependent velocity profile, $v(x,t)$, is then defined in terms of $V_\mathrm{p}(\xi)$ by

$$v(x,t) \;=\; V_\mathrm{p}\left(x - x_\mathrm{p}(t) + \xi_\mathrm{p}\right) \;. \tag{36}$$

Here, $x_\mathrm{p}(t)$ is the advection zone front end position, traveling relative to the stationary x-axis at the constant tip growth velocity, u_p, starting from initial position $x_{\mathrm{p}0}$ at time $t = 0$:

$$x_\mathrm{p}(t) \;=\; \xi_\mathrm{p} \;+\; u_\mathrm{p}\, t \;. \tag{37}$$

The x-coordinate is transformed into the ξ-coordinate here by

$$\xi \;=\; x - x_\mathrm{p}(t) + \xi_\mathrm{p} \;. \tag{38}$$

Note that Eqs. 36 and 37 imply that

$$v(x,t=0) = V_{\rm p}(x)\ . \tag{39}$$

The advection profile shape function, $V_{\rm p}$, has the functional form

$$V_{\rm p}(\xi) \;=\; u_{\rm a}\ f_{v,{\rm lo}}(\xi)\ f_{v,{\rm hi}}(\xi) \tag{40}$$

where $u_{\rm a}$ is the maximal flow velocity inside the advection zone. The dimensionless factors $f_{v,{\rm lo}}(\xi)$ and $f_{v,{\rm hi}}(\xi)$ denote, respectivly, an upward and a downward soft-edge step function which the code implements as piecewise cubics:

$$f_{v,{\rm lo}}(\xi) \;=\; \begin{cases} 0 & \text{for } \xi \;\leq\; \xi_{\rm lo} - W_{v,{\rm lo}}/2 \\ q_{\rm c}\left(\frac{\xi-\xi_{\rm lo}}{W_{v,{\rm lo}}}\right) & \text{for } \xi_{\rm lo} - W_{v,{\rm lo}}/2 \;\leq\; \xi \;\leq\; \xi_{\rm lo} + W_{v,{\rm lo}}/2 \\ 1 & \text{for } \xi_{\rm lo} + W_{v,{\rm lo}}/2 \;\leq\; \xi \end{cases} \tag{41}$$

$$f_{v,{\rm hi}}(\xi) \;=\; \begin{cases} 0 & \text{for } \xi \leq \xi_{\rm hi} - W_{v,{\rm hi}}/2 \\ q_{\rm c}\left(\frac{\xi_{\rm hi}-\xi}{W_{v,{\rm hi}}}\right) & \text{for } \xi_{\rm hi} - W_{v,{\rm hi}}/2 \leq \xi \leq \xi_{\rm hi} + W_{v,{\rm hi}}/2 \\ 1 & \text{for } \xi \geq \xi_{\rm hi} + W_{v,{\rm hi}}/2 \end{cases} \ . \tag{42}$$

Here $q_{\rm c}$ is a cubic polynomial, given by:

$$q_{\rm c}(\beta) \;=\; -2\ \beta^3 \;+\; \frac{3}{2}\ \beta \;+\; \frac{1}{2} \tag{43}$$

where

$$\beta \equiv \frac{\xi - \xi_{\rm lo}}{W_{v,{\rm lo}}}\ , \qquad \text{for} \quad f_{v,{\rm lo}}(\xi)\ , \qquad\qquad \text{and} \qquad\qquad \beta \equiv \frac{\xi_{\rm hi} - \xi}{W_{v,{\rm hi}}}\ , \qquad \text{for} \quad f_{v,{\rm hi}}(\xi)\ . \tag{44}$$

Note that $q_{\rm c}(\beta)$ has the properties

$$q_{\rm c}\Big(-\frac{1}{2}\Big) = 0\ , \quad q_{\rm c}'\Big(-\frac{1}{2}\Big) = 0\ , \quad q_{\rm c}(0) = \frac{1}{2}\ , \quad q_{\rm c}\Big(+\frac{1}{2}\Big) = 1\ , \quad q_{\rm c}'\Big(+\frac{1}{2}\Big) = 0\ . \tag{45}$$

Consequently, $V_{\rm p}(\xi)$ is continuously differentiable everywhere and it takes on the values

$$V_{\rm p}(\xi) \;=\; \begin{cases} 0 & \text{for } \xi \;\leq\; \xi_{\rm lo} - W_{v,{\rm lo}}/2 \\ u_{\rm a}/2 & \text{for } \xi \;=\; \xi_{\rm lo} \\ u_{\rm a} & \text{for } \xi_{\rm lo} + W_{v,{\rm lo}}/2 \;\leq\; \xi \;\leq\; \xi_{\rm lo} - W_{v,{\rm hi}}/2 \\ u_{\rm a}/2 & \text{for } \xi \;=\; \xi_{\rm hi} \\ 0 & \text{for } \xi_{\rm hi} + W_{v,{\rm hi}}/2 \;\leq\; \xi \end{cases} \tag{46}$$

provided that the two edge locations, $\xi_{\rm lo}$ and $\xi_{\rm hi}$ are chosen sufficiently far apart, such that

$$\xi_{\rm hi} - \xi_{\rm lo} \;\geq\; \frac{W_{v,{\rm lo}} + W_{v,{\rm hi}}}{2} \tag{47}$$

The rear and front edge of the advection zone on the ξ-axis are then

$$\xi_{\rm q} \;=\; \xi_{\rm lo} - W_{v,{\rm lo}}/2\ , \qquad\qquad \xi_{\rm p} \;=\; \xi_{\rm hi} + W_{v,+{\rm o}}/2\ . \tag{48}$$

The functions $f_{v,{\rm lo}}(\xi)$, $f_{v,{\rm hi}}(\xi)$ and $V_{\rm p}(\xi)$, plotted *vs.* ξ, are shown below in Figs.S1 and S2.

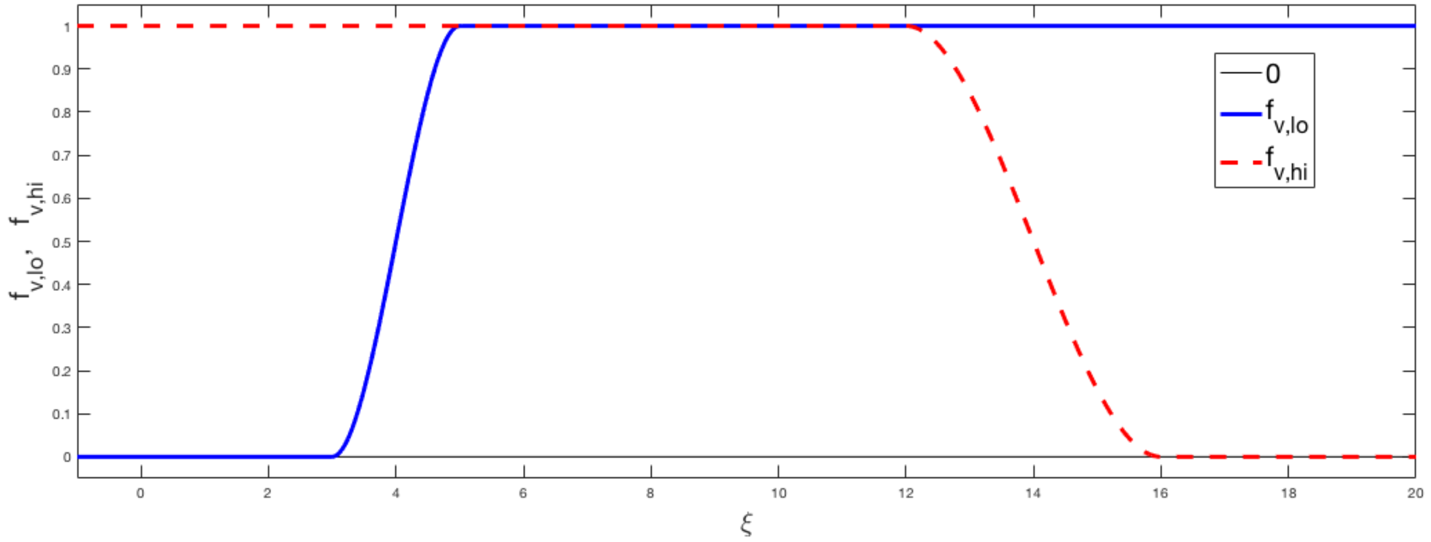

Figure S1. Soft-edge step functions, $f_{v,\mathrm{lo}}(\xi)$ and $f_{v,\mathrm{hi}}(\xi)$, from Option 1 Eqs. 41-44, plotted *vs.* ξ, for edge and width parameters $\xi_{\mathrm{lo}} = 4$, $W_{v,\mathrm{lo}} = 2$, $\xi_{\mathrm{hi}} = 14$, $W_{v,\mathrm{hi}} = 4$.

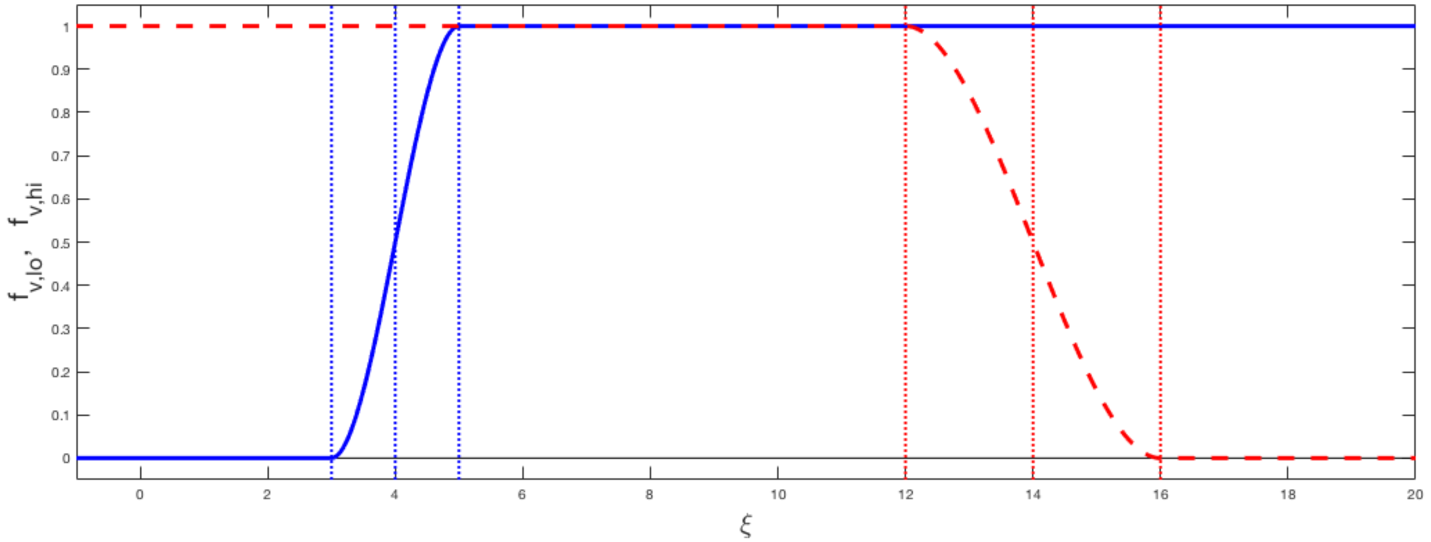

Figure S2. Same as Fig.S1. Vertical dotted lines mark the edge locations and the edge widths, in ascending order, as follows:

blue verticals: $\xi_{\mathrm{lo}} - W_{v,\mathrm{lo}}/2 = 3$, $\xi_{\mathrm{lo}} = 4$, $\xi_{\mathrm{lo}} + W_{v,\mathrm{lo}}/2 = 5$,

red verticals: $\xi_{\mathrm{hi}} - W_{v,\mathrm{hi}}/2 = 12$, $\xi_{\mathrm{hi}} = 14$, $\xi_{\mathrm{hi}} + W_{v,\mathrm{hi}}/2 = 16$,

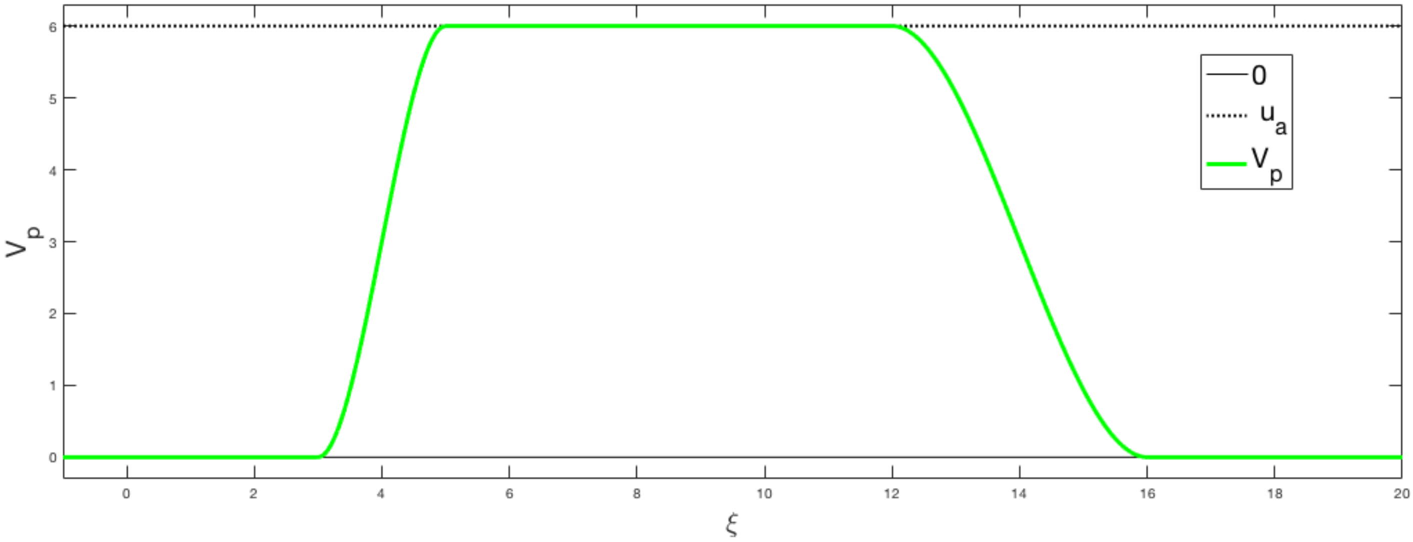

Figure S3. Advection velocity profile shape function,

$$V_\mathrm{p}(\xi) \;=\; u_\mathrm{a}\;\; f_{v,\mathrm{lo}}(\xi)\; f_{v,\mathrm{hi}}(\xi)\;,$$

plotted *vs.* ξ, for Option 1 Eqs. 41-44, and velocity, edge and width parameters

$$u_\mathrm{a} \;=\; 6\;, \quad \xi_\mathrm{lo} \;=\; 4\;, \quad W_{v,\mathrm{lo}} \;=\; 2\;, \quad \xi_\mathrm{hi} \;=\; 14\;, \quad W_{v,\mathrm{hi}} \;=\; 4\;.$$

The edge and width parameters are the same as in Figs.S1 and S2.

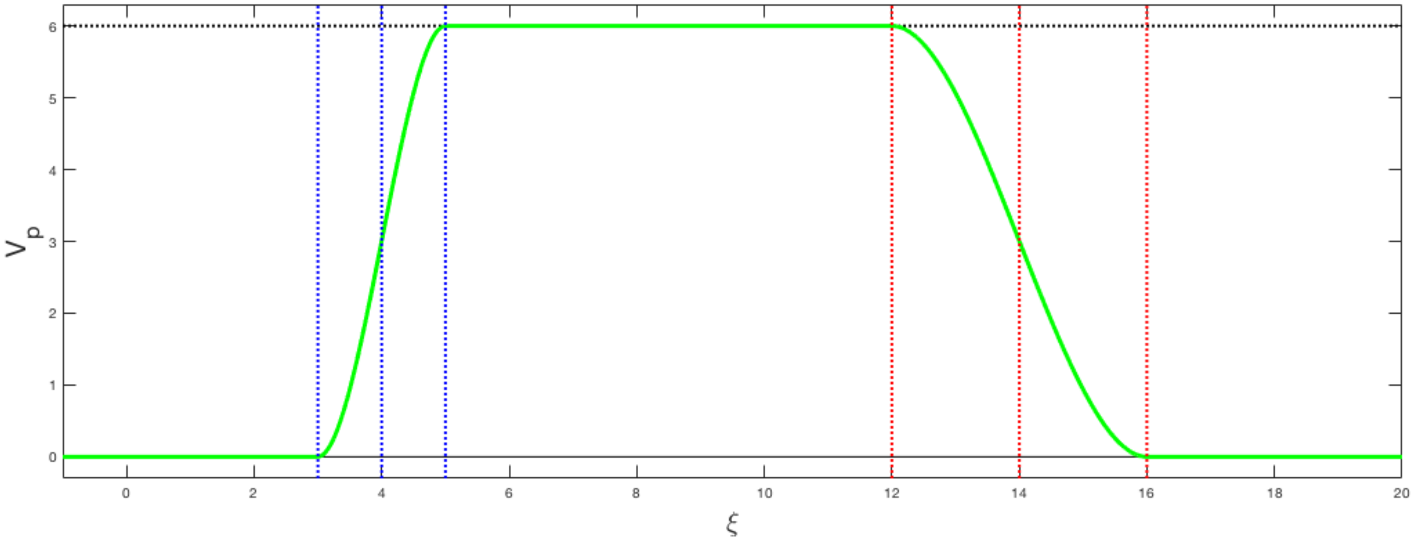

Figure S4. Same as Fig.S3. Vertical dotted lines mark the edge locations and the edge widths, in ascending order, as follows:

blue verticals: $\xi_\mathrm{lo} - W_{v,\mathrm{lo}}/2 \;=\; 3, \quad \xi_\mathrm{lo} \;=\; 4, \quad \xi_\mathrm{lo} + W_{v,\mathrm{lo}}/2 \;=\; 5,$

red verticals: $\xi_\mathrm{hi} - W_{v,\mathrm{hi}}/2 \;=12, \quad \xi_\mathrm{hi} \;=14, \quad \xi_\mathrm{hi} + W_{v,\mathrm{hi}}/2 \;=16,$

Initial Condition (IC) Profile. The PDE system is solved subject to user-supplied initial conditions at $t = 0$, as stated in Eq. 3, assumed to have the form

$$Y_k^{(\text{ini})}(x) \;=\; y_k\, F_{\text{p}}(x) \qquad \text{for species } k = 1, 2, ..., (K-1)\ , \tag{49}$$

excepting the nutrient species, $\Phi(x,t)$, for which we assume a constant, *i.e.*, spatially uniform and time-indepedent extra-cellular supply:

$$Y_K(x,t) \;\equiv\; \Phi^{(\text{ini})} \qquad \text{for all } x \text{ and } t. \tag{50}$$

Here, the y_k denote properly rescaled initial values, mostly derived from the ICs of the single-cell clock model, as described in detail below. The IC profile shape function, $F_{\text{p}}(x)$, determines the spatial variation of the ICs and is assumed to be the same for all species, k. $F_{\text{p}}(x)$ is designed to model an initial inocculation profile in a race tube or in a microdevice channel, with assumed functional form analogous to the $V_{\text{p}}(\xi)$-profile in Eq. 40:

$$F_{\text{p}}(x) \;=\; f_{i,\text{lo}}(x)\ f_{i,\text{hi}}(x)\ . \tag{51}$$

Here, $f_{i,\text{lo}}(x)$ and $f_{i,\text{hi}}(x)$ are, respectively, soft-edge upward and downward step functions, defined in *exactly the same way* as the corresponding piecewise cubic step functions $f_{v,\text{lo}}(\xi)$ and $f_{v,\text{hi}}(\xi)$ in Eqs. 41-44:

$f_{i,\text{lo}}(x)$ is defined by Eq. 41 for $f_{v,\text{lo}}(\xi)$, and $f_{i,\text{hi}}(x)$ is defined by Eq. 42 for $f_{v,\text{hi}}(\xi)$, with following variable name replacements:

$$\xi \;\to\; x\ , \tag{52}$$

$$\xi_{\text{lo}} \;\to\; x_{\text{lo}}\ ,$$

$$W_{v,\text{lo}} \;\to\; W_{i,\text{lo}}\ , \tag{53}$$

$$\xi_{\text{hi}} \;\to\; x_{\text{hi}}\ ,$$

$$W_{v,\text{hi}} \;\to\; W_{i,\text{hi}}\ . \tag{54}$$

B. Rate Functions of Production, Consumption, Advection andd Diffusion

Overview of the PDE Syetem. The PDE system consists of the rate equations for $K = 17$ dynamical variables, $Y_k(x,t)$, representing the $K-6 = 11$ intra-celluar molecular species concentrations, 4 intra-cellular partial nuclear densities and 2 extra-cellular species concentrations. Specifically, the PDE system comprises Eqs. 55 - 64 and 66 for the intra-cellular molecular species concentrations, Eqs. 69 - 72 for the intra-cellular partial nuclear densities, Eq. 67 for the extra-cellular concentrations of the signaling molecule, $[Se]$, and Eq. 75.

All K rate equations of this PDE system, representing both the intra-cellular molecular and nuclear species and the extra-cellular species, are of the general form stated in Eq. 25. Their right-hand sides consist of a reactive rate term, $R_k(\hat{Y}(x,t),t)$, possibly of an advection term, $-\partial_x(v_k(x,t)Y_k(x,t))$ and possibly of a diffusion term, $\Delta_k\,\partial_x^2 Y_k(x,t)$. By definition,

$R_k(\hat{Y}(x,t),t)$, includes all rate contributions which depend only on the concentration values, $Y_k(x,t)$, at location x and time t, but do *not* depend on the x-derivatives of any $Y_k(x,t)$.

The rate equations of this PDE system are augmented by the sum rule Eqs. 17-22 and 9 which allow us to calculate the six gene state densities, frq^0, frq^1, ccg^0, ccg^1, wc–1 and wc–2, as well as the total intra-cellular nuclear density, from the partial nuclear densities. The gene state densities are therefore not treated as separate dynamical variables of the PDE system, but they are required inputs into the transcription rate contributions to $R_k(\hat{Y}(x,t,t)$ of the intra-cellular mRNA species.

Intra-Cellular Molecular Species $k \leq K-6$. The rates of concentration changes of the intra-cellular molecular species $k = 1, ..., K-6$, include the reactive rate functions, $R_k(\hat{Y}(x,t),t)$, and the advective rates, entering into Eq.25. They are given by:

$$\begin{aligned}
\frac{d[wc\text{–}1^{r0}](x,t)}{dt} &= S_1\,[wc\text{–}1^1](x,t) - D_1\,[wc\text{–}1^{r0}](x,t) - C_1\,[wc\text{–}1^{r0}](x,t)\,[FRQ](x,t) \\
&\quad - \partial_x \Big(v(x,t)\ [wc\text{–}1^{r0}](x,t) \Big) \qquad (55)\\
\frac{d[wc\text{–}1^{r1}](x,t)}{dt} &= C_1\,[wc\text{–}1^{r0}](x,t)\,[FRQ](x,t) - D_7\,[wc\text{–}1^{r1}](x,t) \\
&\quad - \partial_x \Big(v(x,t)\ [wc\text{–}1^{r1}](x,t) \Big) \qquad (56)\\
\frac{d[WC\text{–}1](x,t)}{dt} &= L_1\,[wc\text{–}1^{r1}](x,t) \\
&\quad - \Big(D_4 + C_{24}([Si](x,t))\,[WC\text{–}2](x,t) \Big)\,[WC\text{–}1](x,t) \\
&\quad - \partial_x \Big(v(x,t)\ [WC\text{–}1](x,t) \Big) \qquad (57)\\
\frac{d[wc\text{–}2^{r1}](x,t)}{dt} &= S_2\,[wc\text{–}2^1](x,t) - D_2\,[wc\text{–}2^{r1}](x,t) \\
&\quad - \partial_x \Big(v(x,t)\ [wc\text{–}2^{r1}](x,t) \Big) \qquad (58)\\
\frac{d[WC\text{–}2](x,t)}{dt} &= L_2\,[wc\text{–}2^{r1}](x,t) \\
&\quad - \Big(D_5 + C_{24}([Si](x,t))\,[WC\text{–}1](x,t) \Big)\,[WC\text{–}2](x,t) \\
&\quad + P\,([FRQ](x,t))^{m_{\mathrm{F}}}\,[WCC](x,t) \\
&\quad - \partial_x \Big(v(x,t)\ [WC\text{–}2](x,t) \Big) \qquad (59)\\
\frac{d[frq^{r1}](x,t)}{dt} &= S_3\,[frq^0](x,t) + S_4\,[frq^1](x,t) - D_3\,[frq^{r1}](x,t) \\
&\quad - \partial_x \Big(v(x,t)\ [frq^{r1}](x,t) \Big) \qquad (60)\\
\frac{d[FRQ](x,t)}{dt} &= L_3\,[frq^{r1}](x,t) - D_6\,[FRQ](x,t) \\
&\quad - \partial_x \Big(v(x,t)\ [FRQ](x,t) \Big) \qquad (61)\\
\frac{d[WCC](x,t)}{dt} &= C_{24}([Si](x,t))\,[WC\text{–}1](x,t)\,[WC\text{–}2](x,t)
\end{aligned}$$

$$
\begin{aligned}
&- \Big(D_8 + P\left([FRQ](x,t)\right)^{m_{\rm F}}\Big)\,[WCC](x,t) \\
&- \partial_x \Big(v(x,t)\ [WCC](x,t)\Big) \qquad (62)\\
\frac{d[ccg^{r1}](x,t)}{dt} &= S_{\rm c}\,[ccg^1](x,t) - D_{\rm cr}\,[ccg^{r1}](x,t) \\
&- \partial_x \Big(v(x,t)\ [ccg^{r1}](x,t)\Big) \qquad (63)\\
\frac{d[CCG](x,t)}{dt} &= L_{\rm c}\,[ccg^{r1}](x,t) - D_{\rm cp}\,[CCG](x,t) \\
&- \partial_x \Big(v(x,t)\ [CCG](x,t)\Big) \qquad (64)
\end{aligned}
$$

where

$$
C_{24}([Si]) \;=\; \max\Big[\Big(C_2 - C_4\,[Si]\Big), 0\Big]\ . \qquad (65)
$$

By Eq. 65, the intra-cellular signaling species, Si, suppresses WCC-production and shuts it down completely, *i.e.*, $C_{24}([Si]) = 0$, when $[Si]$ exceeds the threshold value $[Si]_{\rm T} = C_2/C_4$. The rate equations for the intra- and extra-cellular signal concentrations, $[Si]$ and $[Se]$, are stated, as part of the PDE system, in Eqs. 66-67 below.

In the foregoing Eqs. 60, and 63, the active and inactive gene state densities, ($[frq^0](x,t)$, $[ccg^0](x,t)$, $[frq^1](x,t)$, $[ccg^1](x,t)$, $[wc\text{–}1^1](x,t)$, $[wc\text{–}2^1](x,t)$) are given in terms of the partial nuclear densities by Eqs. 17-22. The partial nuclear densities are determined, as dynamical variables of the PDE system, by the rate equations stated below in Eqs. 69-72.

Intra- and Extra-Cellular Signaling Species. The rate function $R_{K\text{–}7}$, for the intra-cellular signal, $[Si] \equiv Y_{K\text{–}7}$, includes an intra-cellular signal degradation term, with rate coefficient D_9, and cross-membrane diffusion terms, describing the transport of Si into the extra-cellular volume, and the transport of Se from the extra-cellular into the intra-cellular volume, as given in Ref [3] and used in our earlier work.

$$
\begin{aligned}
\partial_t\,[Si](x,t) \;=\; & k_{\rm Si}\,[CCG](x,t) \;-\; D_9\,[Si](x,t) \;+\; k_{\rm int}\Big([Se](x,t) \;-\; [Si](x,t)\Big) \\
& -\; \partial_x\Big(v(x,t)\ [Si](x,t)\Big)\ . \qquad (66)
\end{aligned}
$$

where $k_{\rm int}$ is the intra-cellular cross-membrane diffusion rate coefficent, denoted by "η" in Ref.[3] $k_{\rm int} \equiv \eta$.

We do *not* employ the quasi-stationary approximation of Ref.[3] for the extra-cellular signal, Se. Rather, $[Se](x,t) \equiv Y_{K\text{–}1}(x,t)$ is treated as a dynamical variable which evolves in space and time, subject to its own PDE. This allows for the possibility of modeling 1D diffusion transport of Se, to occur independently of $[Si]$ in the extra-cellular space, along the filament direction. With a diffusion coefficient Δ_{Se}, the resulting PDE is then given by:

$$
\partial_t\,[Se](x,t) \;=\; -\,D_{10}\,[Se](x,t) \;+\; k_{\rm ext}\Big([Si](x,t) \;-\; [Se](x,t)\Big) \;+\; \Delta_{Se}\,\partial_x^2\,[Se](x,t)\ . \qquad (67)
$$

where $k_{\rm ext}$ is the extra-cellular cross-membrane diffusion rate coefficent, given by

$$
k_{\rm ext} \;=\; \frac{\mathcal{V}_{\rm int}}{\mathcal{V}_{\rm ext}}\ k_{\rm int}\ . \qquad (68)
$$

Here, $\mathcal{V}_{\text{int}}$ and $\mathcal{V}_{\text{ext}}$ denote the total intra-cellular volume and the total extra-cellular volume, respectively. Note that Eq. 67 does *not* contain an advection term, since Se resides outside of the filament and is therefore not subject to transport by the intra-cellular fluid flow.

Nuclear and Nutrient Species. The PDEs for the partial nuclear densities, ρ_{00}, ρ_{01}, ρ_{10} and ρ_{11}, are given by

$$
\begin{aligned}
\partial_t \rho_{00}(x,t) \;=\;& -(A_f + A_c)\,\Omega(x,t)\,\rho_{00}(x,t) \;+\; B_f\,\rho_{10}(x,t) \;+\; B_c\,\rho_{01}(x,t) \\
& + \Gamma(x,t)\,\Big(\rho_{00}(x,t) \;+\; 2\rho_{01}(x,t) \;+\; 2\rho_{10}(x,t) \;+\; 2\rho_{11}(x,t)\Big) \\
& \quad \times\; \Phi(x,t)\,\Big(1-(\rho(x,t)/\rho_{\text{Lim}})^{\epsilon_\rho}\Big)\,\Theta\Big(1-(\rho(x,t)/\rho_{\text{Lim}})^{\epsilon_\rho}\Big) \\
& - \partial_x\Big(r^{(\text{N})} v(x,t)\,\rho_{00}(x,t)\Big) \qquad (69) \\
\partial_t \rho_{01}(x,t) \;=\;& -(A_f\,\Omega(x,t) + B_c)\,\rho_{01}(x,t) \;+\; B_f\,\rho_{11}(x,t) \;+\; A_c\,\Omega(x,t)\,\rho_{00}(x,t) \\
& - \Gamma(x,t)\,\Phi(x,t)\,\rho_{01}(x,t)\,\Big(1-(\rho(x,t)/\rho_{\text{Lim}})^{\epsilon_\rho}\Big)\,\Theta\Big(1-(\rho(x,t)/\rho_{\text{Lim}})^{\epsilon_\rho}\Big) \\
& - \partial_x\Big(r^{(\text{N})} v(x,t)\,\rho_{01}(x,t)\Big) \qquad (70) \\
\partial_t \rho_{10}(x,t) \;=\;& -(B_f + A_c\,\Omega(x,t))\,\rho_{10}(x,t) \;+\; A_f\,\Omega(x,t)\,\rho_{00}(x,t) \;+\; B_c\,\rho_{11}(x,t) \\
& - \Gamma(x,t)\,\Phi(x,t)\,\rho_{10}(x,t)\,\Big(1-(\rho(x,t)/\rho_{\text{Lim}})^{\epsilon_\rho}\Big)\,\Theta\Big(1-(\rho(x,t)/\rho_{\text{Lim}})^{\epsilon_\rho}\Big) \\
& - \partial_x\Big(r^{(\text{N})} v(x,t)\,\rho_{10}(x,t)\Big) \qquad (71) \\
\partial_t \rho_{11}(x,t) \;=\;& -(B_f + B_c)\,\rho_{11}(x,t) \;+\; (A_f\,\rho_{01}(x,t) \;+\; A_c\,\Omega(x,t)\,\rho_{10}(x,t))\,\Omega(x,t) \\
& - \Gamma(x,t)\,\Phi(x,t)\,\rho_{11}(x,t)\,\Big(1-(\rho(x,t)/\rho_{\text{Lim}})^{\epsilon_\rho}\Big)\,\Theta\Big(1-(\rho(x,t)/\rho_{\text{Lim}})^{\epsilon_\rho}\Big) \\
& - \partial_x\Big(r^{(\text{N})} v(x,t)\,\rho_{11}(x,t)\Big)\;. \qquad (72)
\end{aligned}
$$

Here, $\Theta(...)$ denotes the Heaviside step function, *i.e.*, $\Theta(\xi) = 1$ for $\xi > 0$, else $\Theta(\xi) = 0$. The parameter ρ_{Lim} allows to impose an upper limit on the mitotic growth of the nuclear density, independently of nutrient availability.

The factor $\Omega(x,t)$ is shorthand for

$$\Omega(x,t) \;=\; ([WCC](x,t))^{n_{\text{W}}} \qquad (73)$$

and n_{W} is the cooperativity exponent for WCC binding to both the frq and ccg activator binding sites. The factor $\Gamma(x,t)$ is shorthand for

$$\Gamma(x,t) \;=\; k_{\text{ND}} \;+\; k_{\text{DW}}\,([WCC](x,t))^{n_{\text{DW}}} \;+\; k_{\text{DC}}\,([CCG](x,t))^{n_{\text{DC}}}\;. \qquad (74)$$

Here, k_{ND}, k_{DW} and k_{DC} are the rate coeffs for basal (unregulated) nuclear division, WCC-regulated nuclear division and CCG-regulated nuclear division, respectively, with coresponding cooperativity exponents n_{DW} and n_{DC}. For the results reported in this article, we have included only the k_{ND}-term for unregulated divisions. We have not included WCC- or CCG-regulated nuclear division terms, by setting $k_{\text{DW}} = 0$ and $k_{\text{DC}} = 0$.

In Eqs. 69 - 72, we are assuming that each nuclear division results in two daughter nuclei that are both in the "00" gene activation state, as defined in Eq. 4, regardless of the gene state of the parent nucleus. Consequently, the division of a parent nucleus initially in either

the "00", "01", "10" or "11" state, is described by the removal of that parent nucleus and the creation of two daughter nuclei in the "00" state. This is reflected in the stoichiometric pre-factors of the various $\Gamma(x,t)$-terms in Eqs. 69 - 72.

The activation and de-activation of the genes frq and ccg requires the binding and unbinding, respectively, of n_{W} WCC molecules to and from the respective activator binding sites. These processes therefore consume and, respectively, produce free WCC-molecules at the rates proportional to the A_f-, B_f-, A_c- and B_c-terms appearing in Eqs. 69-72. In principle, corresponding rate terms should then also be included in the WCC rate functions, *i.e.*, on the right-hand side of Eq. 62. However, protein molecular concentrations, such as $[WCC]$, (in units of number of molecules per cell volume) are typically on the order of 10^2-10^3 times larger than nuclear densities (in units of number of nuclei per cell volume). Consequently, these (de-)activation-related rates of WCC-consumption and -production are expected to have a negligible effect on the overall WCC-concentration, $[WCC](x,t)$.

We have therefore neglected the gene (de-)activation-related rate terms from the WCC rate equation, Eq. 62. That is, we treat WCC as being "catalytic" in the gene (de-)activation processes, instead of being consumed or produced by these proceses. This approximation simplifies the model in that it allows us to measure molecular concentrations and nuclear densities in two different units, without requiring the conversion factor between these two units as a model input parameter. This is made explicit in the model parameter tabulations shown in Section D.

The extra-cellular nutrient species rate function is governed, in principle, by nutrient consumption due to nuclear proliferation, *i.e.*,

$$\partial_t \Phi(x,t) \;=\; -\,k_\Phi\;\Gamma(x,t)\,\Phi(x,t)\,\rho(x,t)\,\Big(1-(\rho(x,t)/\rho_{\mathrm{Lim}})^{\epsilon_\rho}\Big)\,\Theta\Big(1-(\rho(x,t)/\rho_{\mathrm{Lim}})^{\epsilon_\rho}\Big)\;. \tag{75}$$

Note that the extra-cellular nutrient species is again *not* subject to advection: its rate function in Eq. 75 does not contain the $v(x,t)$-term.

For the results reported in this article, we have actually approximated the nutrient concentration as a spatio-temporal constant, *i.e.*, we have set

$$Y_K(x,t) \;\equiv\; \Phi(x,t) \;\approx\; \Phi^{(\mathrm{ini})} \;=\; \text{constant} \tag{76}$$

This is an accurate approximation if, at least for the duration, T, of the PDE solution time interval, the initial external nutrient supply, $\Phi^{(\mathrm{ini})}$ is far in excess of the total amount of nutrient consumed; and/or if there is a steady chemostatic re-supply of nutrients taking place so as to maintain a constant nutrient level. In this approximation, the initial concentration, $\Phi^{(\mathrm{ini})}$, is substituted for $\Phi(x,t)$ in all rate functions above containing $\Phi(x,t)$.

C. Spatial Discretization and Numerical Time Evolution

Space-Time Grid and Numerical Time Evolution. The PDE system is approximated by an ordinary differential equation (ODE) system, by way of discretizing the x-domain,

with an x-grid defined by

$$x_j \;=\; dx\,(j-1) \qquad \text{for } j = 1,2,...,N_{\rm L}. \tag{77}$$

where $N_{\rm L}$ is the number of x-grid points and and dx is the x-grid spacing:

$$dx \;=\; L/(N_{\rm L}-1)\;. \tag{78}$$

The details of the spatial discretization approximation for the advection and/or diffusion rate terms in Eq. 25 are decribed below. By way of this discretization, the PDE system, Eq. 25, is approximated by a system of ordinary differential equations (ODEs) for the dynamical variables, $Y_k(x_j,t)$, defined on the finite x-grid given by Eq. 77.

The resulting ODE system is then solved numerically by an adaptive $4^{\rm th}$ order Runge-Kutta algorithm[4] with a relative error tolerance of 10^{-6} The solution, $Y_k(x_j,t_\ell)$, for all species, k, is written out for all x-grid points, x_j, on an equidistant grid of output times, t_ℓ, defined by:

$$t_\ell \;=\; dt\,(\ell-1) \qquad \text{for} \quad \ell = 1,2,...,(N_{\rm T}+1). \tag{79}$$

where $N_{\rm T}+1$ is the number of output t-grid points, T is the length of the PDE solution time interval,

$$0 \;\le\; t \;\le\; T\;, \tag{80}$$

and dt is the t-grid spacing:

$$dt \;=\; T/N_{\rm T}\;. \tag{81}$$

Discretization Approximations for Advection and Diffusion Rates. The first and second x-derivatives required for the advection and diffusion rate terms in Eq. 25 are evaluated from the spatially discretized dynamical variables, $Y_k(x_j,t)$, defined on the x-grid, x_j, by the following finite difference approximations:[1]

The first x-derivatives occuring in the advection rate terms in Eqs. 55 - 64 and 66 are approximated by:

$$\partial_x J_k^{\rm (Adv)}(x_j,t) \;\approx\; \frac{1}{2dx}\left(J_k^{\rm (Adv)}(x_{j+1},t) - J_k^{\rm (Adv)}(x_{j-1},t)\right) \tag{82}$$

where $J_k^{\rm (Adv)}(x_j,t)$ is given in terms of $v_k(x_j,t)$ and $Y_k(x_j,t)$ by Eq. 27.

The second x-derivative occuring in the diffusion rate term for Se in Eq. 67 is approximated by

$$\partial_x^2 Y_k(x_j,t) \;\approx\; \frac{1}{dx^2}\left(Y_k(x_{j+1},t) - 2Y_k(x_j,t) + Y_k(x_{j-1},t)\right) \tag{83}$$

The out-of-bounds values of $Y_k(x_{j-1},t)$ for $j=1$ and $Y_k(x_{j+1},t)$ for $j=N_{\rm L}$ are fixed by the boundary conditions

$$Y_k(x_{j-1},t) \;=\; 0 \quad \text{for } j=1, \qquad\qquad Y_k(x_{j+1},t) \;=\; 0 \quad \text{for } j=N_{\rm L}. \tag{84}$$

The foregoing two spatial discretization approximations transform the original PDE model described in Section B into an ODE system for a discrete set of $K \times N_{\rm L}$ dynamical variables,

$Y_k(x_j, t)$ with $k = 1, \ldots, K$ and $j = 1, \ldots, N_{\rm L}$. This ODE system is solved numerically by an adaptive 4$^{\rm th}$ order Runge-Kutta algorithm, as described above.

Controlling the spatial discretization error. To estimate and control the systematic errors introduced by the spatial discretization approximations, Eqs. 82 - 83, we solve the discretized PDE system for a doubling sequence, $N_{\rm L}^{(\iota)}$, of spatial grid sizes, *i.e.*, for $N_{\rm L}^{(\iota)} - 1 = 400, 800, 1600, \ldots$ with doubling sequence index $\iota = 1, 2, 3, \ldots$. We then calculated the relative root mean square (RMS) of the differences between the solutions, $Y_k^{(\iota)}(x,t)$, for successive doubled grid sizes, $N_{\rm L}^{(\iota)}$ and $N_{\rm L}^{(\iota+1)}$, over all x-grid points, $x_j^{(\iota)}$, of the lesser of the two grid sizes, at fixed times t, given by

$$E_k^{(\iota)}(t) = \frac{{\rm RMS}_x^{(\iota)}[Y_k^{(\iota+1)}(x,t) - Y_k^{(\iota)}(x,t)]}{{\rm RMS}_x^{(\iota)}[(Y_k^{(\iota+1)}(x,t) + Y_k^{(\iota)}(x,t))/2]} \; . \tag{85}$$

For any function $Y(x,t)$ defined on the x-domain $[0, L]$ and for any time t, the RMS-value, ${\rm RMS}_x^{(\iota)}[Y(x,t)]$, is defined here by

$${\rm RMS}_x^{(\iota)}[Y(x,t)] = \sqrt{\frac{1}{N_{\rm L}^{(\iota)}} \sum_{j=1}^{N_{\rm L}^{(\iota)}} |Y(x_j^{(\iota)}, t)|^2} \tag{86}$$

where, by Eqs. 77 and 78, and

$$x_j^{(\iota)} = \frac{L}{N_{\rm L}^{(\iota)} - 1} (j-1) \; . \tag{87}$$

Based on the foregoing relative error estimation, we chose to report in the main text of this article the PDE solution results for the grid size $N_{\rm L}^{(\iota)} = 3201$ with a relative error of $E_k^{(\iota-1)}(t) < 5\%$ for the clock species FRQ up to time $t = 200\,{\rm h}$.

D. Model Parameters

The following tables summarize the commplete set of parameters that were used to produce the PDE model results reported in this article. Molecular concentrations are measured in *Molecular Concentration Units* (mcu). Nuclear densities and related gene state densities are measured in *Nuclear Density Units* (ndu). Times are measured in units of hours (h). Lengths are measured in units of millimeters (mm).

Table 1: Non-zero rate coefficients and related parameters of the PDE model defined by Eqs. 55 - 76. Any parameters occuring in the foreoging equations that are not listed in the tabulation below have been set to zero. In addition, intra-cellular flow and advection velocity parameters entering into Eqs. 29, 31, 37 and 40, *i.e.*, the parameters $r^{(\mathrm{N})}$, u_p and u_a, are listed below. Units of molecular concentration, nuclear density, time, and length, denoted by mcu, ndu, h and mm, are explained in text above.

Name	Value	Unit	Name	Value	Unit
n_W	4	1	m_F	4	1
A_f	7.0786×10^{-11}	$\mathrm{mcu}^{-n_\mathrm{W}} \cdot \mathrm{h}^{-1}$	B_f	0.10131	h^{-1}
A_c	2.7435×10^{-8}	$\mathrm{mcu}^{-n_\mathrm{W}} \cdot \mathrm{h}^{-1}$	B_c	9.5278	h^{-1}
S_1	0.28495	$(\mathrm{mcu}/\mathrm{ndu}) \cdot \mathrm{h}^{-1}$	S_2	0.0033487	$(\mathrm{mcu}/\mathrm{ndu}) \cdot \mathrm{h}^{-1}$
S_3	0.0017219	$(\mathrm{mcu}/\mathrm{ndu}) \cdot \mathrm{h}^{-1}$	S_4	0.19483	$(\mathrm{mcu}/\mathrm{ndu}) \cdot \mathrm{h}^{-1}$
S_c	0.59707	$(\mathrm{mcu}/\mathrm{ndu}) \cdot \mathrm{h}^{-1}$	P	24.613×10^{-11}	$\mathrm{mcu}^{-m_\mathrm{F}} \cdot \mathrm{h}^{-1}$
L_1	4234.5	h^{-1}	L_2	2089.6	h^{-1}
L_3	5.2833	h^{-1}	L_c	12.560	h^{-1}
D_1	1.1955	h^{-1}	D_2	1.0000	h^{-1}
D_3	0.47631	h^{-1}	D_4	0.54289	h^{-1}
D_5	0.37079	h^{-1}	D_6	0.22669	h^{-1}
D_7	0.043017	h^{-1}	D_8	6.5869×10^{-5}	h^{-1}
D_9	21.505	h^{-1}	D_{10}	1.2145×10^{-5}	h^{-1}
D_cr	60.4984500	h^{-1}	D_cp	0.39410	h^{-1}
C_1	1.6854	$(\mathrm{mcu} \cdot \mathrm{h})^{-1}$	C_2	0.036126	$(\mathrm{mcu} \cdot \mathrm{h})^{-1}$
C_4	0.026678	$(\mathrm{mcu}^2 \cdot \mathrm{h})^{-1}$	k_Si	9.2256	h^{-1}
$k_\mathrm{ext}/k_\mathrm{int}$	1.00000	1	k_int	0.0057831	h^{-1}
$k_\mathrm{N}\, \Phi^{(\mathrm{ini})}$	2.0000	h^{-1}	ρ_Lim	4.00000	ndu
$r^{(\mathrm{N})}$	0.357479	1	ϵ_ρ	5	1
u_p, u_a	0.388722	mm/h			

Table 2: Size and shape parameters of the drift velocity profile, and of the inocculated initial concentration and density profiles, entering into Eqs. 36 - 47 and Eqs. 51 - 54 respectively. The advection zone front and rear coordinates, ξ_{p} and ξ_{q}, are not listed below, since they can be obtained from the drift velocity profile parameters below by Eq.48.

Name	Value	Unit	Name	Value	Unit
ξ_{lo}	20.0000	mm	ξ_{hi}	45.0000	mm
$W_{v,\mathrm{lo}}$	4.00000	mm	$W_{v,\mathrm{hi}}$	4.00000	mm
x_{lo}	20.0000	mm	x_{hi}	45.0000	mm
$W_{i,\mathrm{lo}}$	4.00000	mm	$W_{i,\mathrm{hi}}$	4.00000	mm

Table 3: Initial inocculation concentration parameters, y_k, for all dynamical variable species, $k \leq (K-1)$, with non-zero initial concentration (IC), $Y_k^{(\text{ini})}(x) > 0$, inside the inocculation zone interval $(x_{\text{lo}} - W_{i,\text{lo}}/2,\ x_{\text{hi}} + W_{i,\text{hi}}/2)$, as defined in Eqs. 49 - 54. By Eqs. 49 and 41 - 44, y_k is the maximum concentration value, attained by $Y_k^{(\text{ini})}(x)$ in the interior of the inocculation zone interval, $[x_{\text{lo}} + W_{i,\text{lo}}/2,\ x_{\text{hi}} - W_{i,\text{hi}}/2]$, for the initialization profile parameters listed in Table 2. The extracellular nutrient species concentration, $Y_K(x,t)$, is assumed to be a constant, $\Phi^{(ini)}$, for all x and t by Eq. 50, and is therefore excluded from this table. For all species with $k \leq (K-1)$ that are not listed in this table, the initial concentration, $Y_k^{(\text{ini})}(x)$, was set zero for all x, and hence y_k=0. This includes all molecular species. Only the partial nuclear densities (ρ_{00}, ρ_{01}, ρ_{10}, ρ_{11}) were given non-zero initial concentrations and are therefore listed below. Their respective dependents, the gene state and total nuclear densities ($[frq^0]$, $[frq^1]$, $[ccg^0]$, $[ccg^1]$, $[wc{-}1^1]$, $[wc{-}2^1]$, ρ), are initialized from these partial nuclear densities, by Eqs. 17 - 22 and Eq. 9, and are therefore also not listed below. The partial nuclear densities, and their dependents, are measured in "ndu" which stands for "Nuclear Density Unit".

Species	y_k	Unit	Species	y_k	Unit
ρ_{00}	0.0441768	ndu	ρ_{01}	0.00484935	ndu
ρ_{10}	0.000486924	ndu	ρ_{11}	0.000486924	ndu

Table 4: Space-time domain size and grid size parameters, as introduced in Eqs. 1,2 and 77 - 81

Name	Value	Unit	Name	Value	Unit
L	200.000	mm	N_L	3201	1
T	240.000	h	N_T	240	1

E. References Cited

[1] A. Sharma, *Introduction to Computational Fluid Dynamics: Development, Application and Analysis*, Springer Nature (2021).

[2] T. L. Bergman, *Fundamentals of Heat and Mass Transfer*, Wiley (2011).

[3] J. Garcia-Ojalvo, M. B. Elowitz, and S. H. Strogatz, Modeling a synthetic multicellular clock: repressilators coupled by quorum sensing, Proc. Natl. Acad. Sci. U.S.A., **101**, 10955 (2004).

[4] W. H. Press, *Numerical Recipes: The Art of Scientific Computing*, 3rd Edition, Cambridge University Press (2007).
